# STZ-induced hyperglycemia differentially influences mitochondrial distribution and morphology in the habenulointerpeduncular circuit

**DOI:** 10.3389/fncel.2024.1432887

**Published:** 2024-12-23

**Authors:** Mohammad Jodeiri Farshbaf, Taelor A. Matos, Kristi Niblo, Yacoub Alokam, Jessica L. Ables

**Affiliations:** ^1^Nash Family Department of Neuroscience, Icahn School of Medicine at Mount Sinai, Friedman Brain Institute, New York, NY, United States; ^2^Department of Psychiatry, Icahn School of Medicine at Mount Sinai, New York, NY, United States; ^3^PREP Program, Icahn School of Medicine at Mount Sinai, New York, NY, United States; ^4^St. Francis College, New York, NY, United States; ^5^Icahn School of Medicine at Mount Sinai, Diabetes Obesity Metabolism Institute, New York, NY, United States

**Keywords:** diabetes, mitochondrial homeostasis, lipid, medial habenula, interpeduncular nucleus

## Abstract

**Introduction:**

Diabetes is a metabolic disorder of glucose homeostasis that is a significant risk factor for neurodegenerative diseases, such as Alzheimer’s disease, as well as mood disorders, which often precede neurodegenerative conditions. We examined the medial habenulainterpeduncular nucleus (MHb-IPN), as this circuit plays crucial roles in mood regulation, has been linked to the development of diabetes after smoking, and is rich in cholinergic neurons, which are affected in other brain areas in Alzheimer’s disease.

**Methods:**

This study aimed to investigate the impact of streptozotocin (STZ)-induced hyperglycemia, a type 1 diabetes model, on mitochondrial and lipid homeostasis in 4% paraformaldehyde-fixed sections from the MHb and IPN of C57BL/6 J male mice, using a recently developed automated pipeline for mitochondrial analysis in confocal images. We examined different time points after STZ-induced diabetes onset to determine how the brain responded to chronic hyperglycemia, with the limitation that mitochondria and lipids were not examined with respect to cell type or intracellular location.

**Results:**

Mitochondrial distribution and morphology differentially responded to hyperglycemia depending on time and brain area. Six weeks after STZ treatment, mitochondria in the ventral MHb and dorsal IPN increased in number and exhibited altered morphology, but no changes were observed in the lateral habenula (LHb) or ventral IPN. Strikingly, mitochondrial numbers returned to normal dynamics at 12 weeks. Both blood glucose level and glycated hemoglobin (HbA1C) correlated with mitochondrial dynamics in ventral MHb, whereas only HbA1C correlated in the IPN. We also examined lipid homeostasis using BODIPY staining for neutral lipids in this model given that diabetes is associated with disrupted lipid homeostasis. BODIPY staining intensity was unchanged in the vMHb of STZ-treated mice but increased in the IPN and VTA and decreased in the LHb at 12 weeks. Interestingly, areas that demonstrated changes in mitochondria had little change in lipid staining and vice versa.

**Discussion:**

This study is the first to describe the specific impacts of diabetes on mitochondria in the MHb-IPN circuit and suggests that the cholinergic MHb is uniquely sensitive to diabetesinduced hyperglycemia. Further studies are needed to understand the functional and behavioral implications of these findings.

## Introduction

Diabetes is one of the most prevalent chronic metabolic diseases. In 2019, more than 37 million Americans had diabetes, which represents a staggering 11.3% of the US population. The prevalence of diabetes continues to grow worldwide, with 1.5 million new cases each year in the United States alone (National Diabetes Statistics Report, n.d.). New therapies and technology over the last two decades have resulted in people living longer with diabetes than ever before. As a result, there is growing evidence that persistent hyperglycemia, the hallmark of diabetes, leads to multiple neurocognitive and neuropsychiatric sequelae, including dementia and mood disorders (Frank, 2005; Biessels and Despa, 2018). Physiological and cellular responses to diabetes and hyperglycemia vary depending on the organ examined (Yin et al., 2021; Messeri et al., 2012), yet there are surprisingly little data on the molecular and cellular sequelae of diabetes in the brain.

Epidemiological data indicate that diabetes is a risk factor for Alzheimer’s disease (AD) (Stanciu et al., 2020), which is characterized by cholinergic neuron dysfunction and death (Ferreira-Vieira et al., 2016), and animal models suggest that cholinergic neurons in the hippocampus are the most susceptible population to persistent hyperglycemia (Welsh and Wecker, 1991; Wang et al., 2009). The habenula is a bilateral nucleus of the epithalamus and is divided into medial (MHb) and lateral (LHb) nuclei. Structure, connection, and gene expression are different in those regions, but they have some related functions at the behavioral level for regulating mood (Aizawa et al., 2012; Ables et al., 2023). The LHb has direct outputs to monoaminergic areas, including the serotoninergic raphe nuclei and the dopaminergic substantia nigra pars compacta (SNc) and ventral tegmental area (VTA) (Namboodiri et al., 2016), while the MHb sends a single projection through the fasciculus retroflexus (FR) to the interpeduncular nucleus (IPN) in the midbrain, which in turn sends projections to other midbrain nuclei to regulate levels of serotonin (5-HT), dopamine (DA), and epinephrine (Xu et al., 2018; Carlson et al., 2001). Furthermore, both habenular nuclei have links with metabolism. For example, habenula lesions increased the sensitivity to insulin in diabetic rats (Qu et al., 2020). Moreover, it has been shown that hyperglycemia increased LHb activity which led to depressive-like behavior in diabetic rats (Dong et al., 2023). Studies have shown that the ventral cholinergic MHb has a crucial role in linking nicotine addiction to diabetes, and in regulating mood-related behaviors, especially those with negative valence (e.g., fear and anhedonia) (Hsu et al., 2016; Zhang et al., 2016; Duncan et al., 2019), which are often found early in the course of AD. Of interest for this study, the ventral portion of the MHb is a cholinergic nucleus, and we hypothesized that it is potentially vulnerable to diabetes-associated hyperglycemia, as are cholinergic neurons in the hippocampus; however, the impact of diabetes on the habenula at the cellular level is totally unknown.

Diabetes leads to differential metabolic changes depending on the organ examined. Mitochondria are the first line of response to metabolic changes in most organs, including the central nervous system (CNS). For example, T1D leads to depolarized mitochondrial membrane potential in dorsal root ganglia (DRG) neurons (Huang et al., 2003). Mitochondrial reactive oxygen species (ROS) are increased in parallel with complex I activity in rat brains with T1D (Silva-Rodrigues et al., 2020). Mitochondria are dynamic organelles and maintaining their correct morphology is crucial for cell function, as mitochondrial morphology correlates with their function and energetic efficiency (Chen and Chan, 2005; Garcia et al., 2019; Lowell and Shulman, 2005; Siegmund et al., 2018). *In vitro* studies demonstrate that mitochondria are fragmented in high glucose concentrations and generate more ROS (Yu et al., 2006; Tien et al., 2017), while glucose deprivation leads to elongated and hyperfused mitochondria in fibroblasts (Lee et al., 2014). In cholinergic neurons, bioenergetic failure of mitochondria is associated with less acetylcholine (ACh) synthesis, highlighting their potential vulnerability in diabetes. Mitochondria generate acetyl-coenzyme A (acetyl-CoA), which is a precursor to ACh synthesis (Szutowicz et al., 2013). Consistent with diabetes as a risk factor, several reports found cholinergic neurons were susceptible to hyperglycemia in AD (Wang et al., 2009; Bell et al., 2006; Agrawal and Agrawal, 2022). Similarly, depression is associated with differential metabolic disturbances depending on the brain area examined (Germain et al., 2007). It was shown that both the MHb and LHb had increased metabolism and mitochondrial cytochrome oxidase activity in helpless rats, a model of depression (Shumake et al., 2003). Therefore, analysis of mitochondrial distribution and network in the habenula in a model of T1D can reveal the impact of chronic hyperglycemia on mitochondrial dynamics in these previously unexamined regions and provide a foundation to link metabolism with mood and risk for neurodegenerative disorders.

Diabetes is also associated with profound disturbances in fat metabolism, as insulin is the primary fat-storage hormone. The brain is the second most lipid-rich organ after adipose tissue. Mitochondrial lipid metabolism supports energy homeostasis in the brain, with some studies estimating that 20% of the energy requirement in the brain is fueled by lipid *β*-oxidation (Ebert et al., 2003). Different types of lipids are present in the brain and change in response to pathological conditions, including diabetes (Yoon et al., 2022). For example, cholesterol metabolism is decreased in the hypothalamus in T1D (Suzuki et al., 2010). Lipid droplet (LD) formation is associated with the energetic status of the cell and can be assessed histologically. During energy shortage, cells shift from glucose to mitochondrial *β*-oxidation to maintain ATP levels (Gerhart-Hines et al., 2007). Pathological conditions such as stroke and neurodegenerative diseases change LD formation, turnover, and localization in the brain (Yang et al., 2014; Brekk et al., 2020; Gasparovic et al., 2001). Interestingly, the MHb is capable of lipid uptake and displays highly active lipid metabolism compared to LHb (Hofmann et al., 2017). Moreover, single-cell transcriptomics showed that mitochondrial genes and fatty acid binding protein 5 (*Fabp5*) had high expression in MHb and LHb (Wallace et al., 2020). While glucose metabolism is altered in these disease states, it is unclear how lipid distribution in the habenula can be influenced by chronic hyperglycemia in T1D.

Given the links between diabetes and depression and subsequent risk for AD and the unique metabolism within the MHb-IPN and LHb-VTA, we investigated the effects of chronic hyperglycemia on mitochondrial homeostasis in MHb-IPN and examined the adjacent LHb and VTA regions to determine whether any observed changes were specific to this circuit. Furthermore, given that diabetes is a chronic condition, we evaluated how dynamic the longitudinal effects of chronic hyperglycemia might be by examining two endpoints after STZ injection: 6 and 12 weeks. Because T1D and the STZ model are associated with derangements in other pancreatic hormones in addition to insulin, we sought to understand if glycemia itself was a driver of the observed changes by correlating blood glucose with mitochondrial distribution and network in the MHb. Finally, we examined the neutral lipid distribution in the MHb-IPN and LHb-VTA over time to evaluate how T1D influenced lipid homeostasis. Our findings reveal previously unappreciated temporal dynamics of mitochondria in response to hyperglycemia and support the premise that cholinergic neurons may be more sensitive to the dysregulation of glucose homeostasis.

## Methods and materials

### Animals

Seven-week-old male C57BL/6 J wild-type mice were purchased from Jackson laboratories (Stock # 000664) and housed five per cage with *ad libitum* access to water and to regular chow (LabDiet 5,053; protein 21%, fat 6%, carbohydrate 53.5%, fiber 4.4%, ash 6%, 4.11 kcal/gm) in ventilated cages. The animal facility room was maintained within a constant temperature (25°C) and humidity (55%) on a regular 12-h light/12-h dark cycle. Experiments were approved by the Mount Sinai Institutional Animal Care and Use Committee and adhered to the National Institutes of Health (NIH) guidelines. Mice were acclimated to the facility for 1 week prior to use.

### Streptozotocin-induced diabetes model

At 8 weeks of age, mice received a daily intraperitoneal injection of either 1x Hank’s balanced saline solution (HBSS) as the vehicle (VEH) control or streptozotocin (STZ, 50 mg/kg) for 5 consecutive days using a modified low-dose protocol. All mice were fasted for 4 h prior to STZ or VEH injection (Furman, 2021; Deeds et al., 2011). STZ was purchased from Sigma (Cat # S0130) and dissolved in HBSS immediately before injection. HBSS (pH 7.4) was used instead of citrate buffer (pH 4.5) to avoid acidosis. To monitor response to STZ, body weight was recorded and a 4-h fasting glucose was measured using a Bayer Contour® Glucometer at baseline and weekly following STZ treatment. To evaluate chronic glycemia, glycated hemoglobin (HbA1c) was measured at sacrifice using the A1CNow + ® SelfCheck Kit (PTS Diagnostics). Animals with 4-h fasting glucose higher than 200 mg/dL were considered diabetic. In total, four cohorts of male mice were used in this study, two cohorts at each time point, with equal numbers of VEH- and STZ-treated mice included in each cohort.

### Immunohistochemistry and histology

Mice were deeply anesthetized by isoflurane and transcardially perfused with 0.1 M phosphate-buffered saline (PBS), pH 7.4, followed by 4% paraformaldehyde (PFA) in 0.1 M PBS, pH 7.4. The whole brain was removed and post-fixed in 4% PFA overnight at 4°C. The brain was kept in a cryoprotective solution (18% glycerol in 0.1 M PBS) until sectioning. We used a sliding freezing microtome to make 40 μm coronal sections of the brains. The sections were kept in cryobuffer (25% glycerol and 25% ethylene glycol in 0.1 M PBS) in 12-well culture plates at −20°C until use. Immunohistochemistry was performed in two batches at each time point. Free-floating sections were washed with PBS two times and then blocked with 3% normal donkey serum and 0.3% Triton X-100 in 0.1 M PBS for 60 min at room temperature. For immunofluorescence, the sections were incubated with primary antibody against TOM-20 (D8T4N, Rabbit mAb, 1:200, Cell Signaling Technology Cat# 42406, RRID:AB_2687663) overnight at 4°C. Following incubation with the primary antibody, the sections were washed with 0.1 M PBS three times for 10 min each at room temperature. The sections were incubated with the Alexa 647-conjugated secondary antibody (donkey anti-rabbit 1:350, Jackson ImmunoResearch, RRID:AB_2892164), for 3 h at room temperature. After three washes (5 min each) with 0.1 M PBS, we incubated sections with diluted BODIPY^493/503^ (1:500 from 1 mg mL^−1^ stock solution in dimethyl sulfoxide (DMSO), Invitrogen#D3922) in 1xPBS for 30 min at room temperature. Sections were washed three times with 0.1 M PBS, mounted on glass slides, coverslipped with Fluoromount-G mounting medium (Invitrogen#00-4958-02), and imaged with a confocal laser scanning microscope (Zeiss LSM 780). VEH- and STZ-treated sections were matched in terms of time in cryoprotective and cryobuffer solutions.

### Confocal imaging

Imaging acquisition of the sample was performed with a Zeiss LSM 780 confocal microscope. We used a Z-stack with an interval of 0.35 μm and a maximum of 30 optical sections. We used objective lenses 10×, 40×, and 63× with different zooms to optimize the signal-to-noise ratio. To analyze mitochondria and LDs, we used 63× objective with zoom 1. Imaging parameters such as laser power, exposure, and pinhole were set up on the VEH group in each cohort and held consistent between specimens.

### Quantification of mitochondrial morphology and network

Mitochondrial morphology and network were performed on the confocal-acquired images using ImageJ software as described (Jayashankar et al., 2021). Briefly, background subtraction and auto threshold were applied to all images. Noise was reduced with the despeckle function from maximum projections of Z-stacks. The ‘Analyze Particles’ function was set with a size range of 0 to infinity (pixel^2) and a circularity range of 0 to 1. To analyze the network, we used the Mitochondria Analyzer plugin in ImageJ to measure the average of networking parameters. Briefly, after background subtraction and noise despeckle, we evaluated images with Mitochondria Analyzer, 2D analysis, in ImageJ. In 2D analysis, we performed per-cell base/per-mito morphologic descriptors, which included mean aspect ratio and form factors. This method was adopted from a published protocol for multidimensional confocal analysis of mitochondrial morphology and dynamics (Chaudhry et al., 2020). We used three to five randomly selected high-power visual fields per animal per structure. Raw values were normalized to the average of the VEH group within each batch of staining.

### Quantification of lipid droplets

We used ImageJ to quantify spherical particles stained with BODIPY as a proxy for lipid droplet quantification. We used maximum projections of Z-stacks with a 1 μm interval for quantification. After removing the background and enhancing the contrast, we removed the noise. Then, we applied Gaussian blur (radius = 1) and then adjusted the threshold based on the control group. We used the same threshold for all images and used pixel sizes 2 to 100 and circularity from 0.7 to 1 to remove cell membrane staining from the analysis. We selected eight regions of interest (ROI) for each field of view. For quantification, three to five sections per mouse were used and three to five images per mouse were averaged.

### Data normalization

ImageJ outputs were averaged across three to five sections to generate a single data point per animal. These values were then normalized to the average of the VEH group within each batch of staining.

### Statistical analysis

All statistical analysis was performed using GraphPad Prism 8. Statistical significance between groups was determined by two-way analysis of variance (ANOVA) followed by a post-hoc Tukey’s test. The normality of the datasets was evaluated via examination of residuals following ANOVA or Shapiro–Wilk test in GraphPad Prism. Detailed information about the number of slices and mice per group for each experiment is indicated in figure legends. All *p*-values are listed in the text and figures. The data are presented as the average ± SEM.

## Results

### STZ treatment induces chronic hyperglycemia as a mouse model of type 1 diabetes

**Figure 1:**
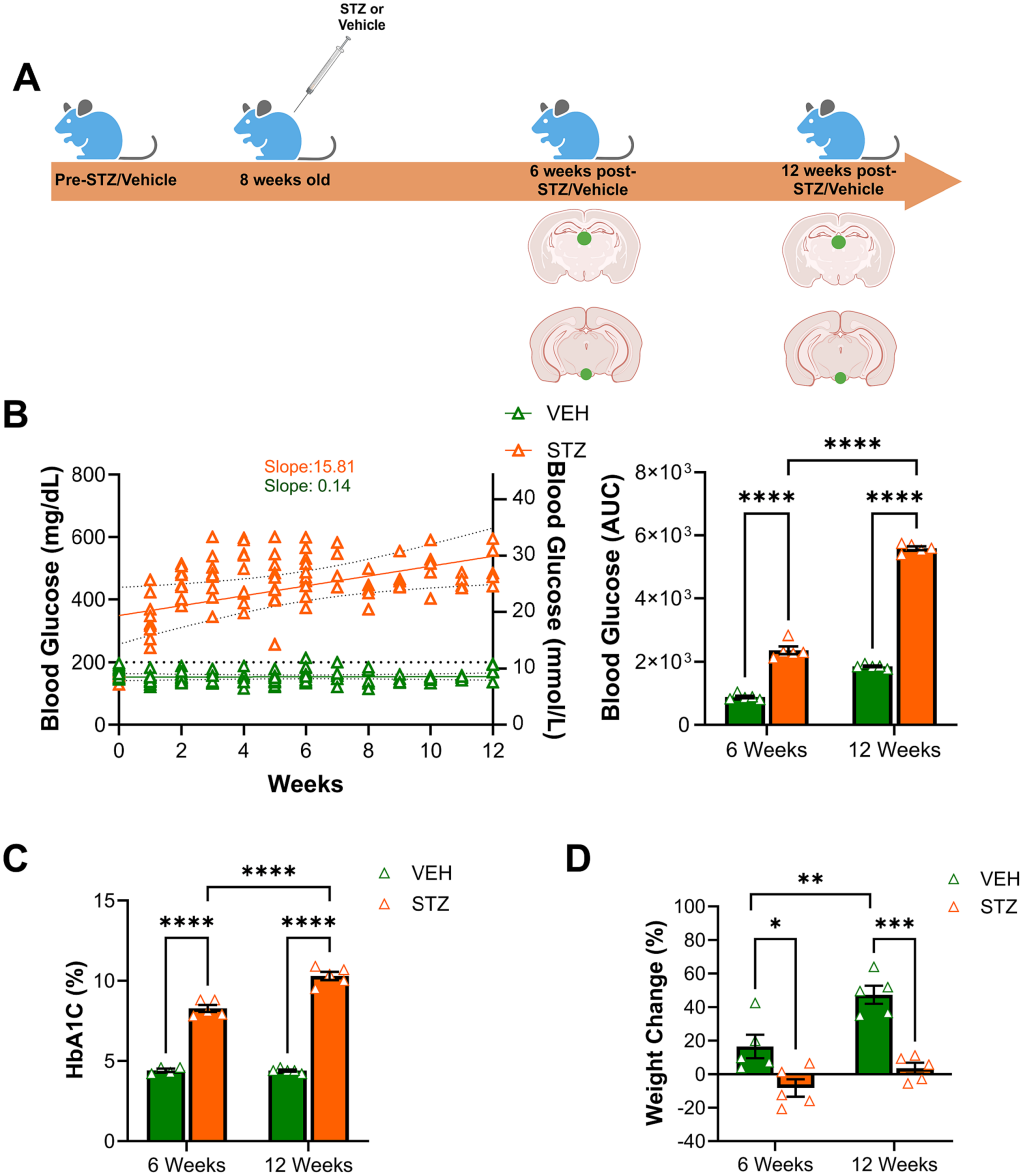
STZ treatment induced chronic hyperglycemia in a mouse model of type 1 diabetes. **(A)** Experimental timeline. 8-week-old male C57BL/6 J 8 mice were injected with Hank’s balanced saline solution (HBSS) as vehicle (VEH) group or streptozotocin (STZ, 50 mg/kg) for 5 consecutive days. Fasting blood glucose and body weight were monitored weekly before and after treatment. At 6- and 12-week endpoints, HbA1C (%) was measured. Mice were perfused, and the brain was removed at 6- and 12-week endpoints for immunohistochemistry. Green dots on the brain sections indicate areas examined, the habenula in the epithalamus, and the IPN at the ventral midbrain. **(B)** Fasting blood glucose (mg/dl on the left axis and equivalent mmol/L on the right axis) was measured weekly. Corresponding area under the curve (AUC) of blood glucose. **(C)** HbA1C (%) was measured at 6- and 12-week endpoints to confirm chronic hyperglycemia. **(D)** Percentage of weight change was calculated at the end of 6- and 12-week endpoints. All statistical analyses were performed using two-way ANOVA followed by Tukey’s *post-hoc* test. N = 5 per group. All results are given as mean ± SEM (*p < 0.05, **p < 0.01, ***p < 0.005, ****p < 0.0001).

To generate a mouse model of insulin-deficient diabetes, which recapitulates aspects of type 1 diabetes, 8-week-old C57BL/6 J male mice were treated with STZ (50 mg/kg, i.p.) for 5 consecutive days. The vehicle group was injected with vehicle (HBSS) in parallel (Figure 1A). Fasting (4 h) blood glucose was measured weekly from the tail vein to monitor glycemia. Otherwise, the mice were group-housed with *ad libitum* access to regular chow and water until perfusion with 4% PFA at either 6-week or 12-week endpoints. As expected, we found that STZ treatment induced rapid and chronic hyperglycemia, lasting until the end of the study (slope_VEH_ = 0.14 and slope_STZ_ = 15.81). Blood glucose level was significantly increased at both of our primary endpoints, 6 and 12 weeks after STZ injection, compared to the VEH group as measured by area under the curve [AUC ((mg/dL) × weeks)] (6 weeks: VEH = 882.40 ± 41.82, STZ = 2366.00 ± 120.96; 12 weeks: VEH = 1855.20 ± 27.33, STZ = 5589.20 ± 62.11; treatment *X* time: *F*_(1,16)_ = 241.3, *p* < 0.0001, effect of treatment: *F*_(1,16)_ = 1,297, *p* < 0.0001, effect of time: *F*_(1,16)_ = 838.9, *p* < 0.0001) (Figure 1B). We also measured HbA1C% to confirm the diabetic phenotype and gauge the level of chronic hyperglycemia at the 6- and 12-week experimental endpoints. We found STZ-treated mice showed significantly higher HbA1C than the VEH-treated group at both 6- and 12-week endpoints (6 weeks: VEH = 4.4 ± 0.10%, STZ = 8.28 ± 0.21%; 12 weeks: VEH = 4.40 ± 0.06%, STZ = 10.30 ± 0.25%; treatment *X* time: *F*_1,15_ = 27.75, *p* < 0.0001, effect of treatment: *F*_1,15_ = 695.2, *p* < 0.0001, effect of time: *F*_(1,15)_ = 29.08, *p* < 0.0001). Consistent with chronic and persistent hyperglycemia in the STZ-treated mice, HbA1C was significantly higher at the 12-week endpoint (10.30 ± 0.25%) than at the 6-week endpoint (8.28 ± 0.21%) (Figure 1C). Consistent with previous reports using the STZ model (Talbot et al., 2024; Nørgaard et al., 2018; Graham et al., 2011), male C57BL/6 J mice lost weight 6 weeks post-STZ injection (−8.25 ± 5.19% baseline weight), although the difference in absolute weight between groups was not significant (*t-*test: T_8_ = 1.35, *p* = 0.21). At the 12-week endpoint, the STZ group showed less weight gain (3.56 ± 3.30% baseline weight) compared to the VEH group (6 weeks: VEH = 16.53 ± 7.02%, STZ = -8.25 ± 5.19%; 12 weeks: VEH = 47.33 ± 5.38%, STZ = 3.56 ± 3.30%; effect of treatment: *F*_(1,16)_ = 40.41, *p* < 0.0001, effect of time: *F*_(1,16)_ = 15.62, *p* = 0.001) (Figure 1D). In the STZ mice, there was not a significant difference in weight change compared to the 6-week endpoint (*p* = 0.43) although most mice returned to baseline weight. Their body weight before and after treatment (VEH and STZ) is listed in Table 1. Overall, we found that STZ-induced robust and chronic hyperglycemia in male mice, with modest weight loss in the first phase (6w) followed by recovery to baseline (12w).

**Table 1:** Bodyweight before and after STZ/VEH treatment.

Treatment	Bodyweight (g)/ Pre-treatment	Bodyweight (g)/ 6-week endpoint	Bodyweight (g)/ pre-treatment	Bodyweight (g)/ 12-week endpoint
Vehicle				
	21.2	30.2	24.3	32.2
	22.0	22.8	22.6	36.9
	24.6	29.5	25.5	33.8
	31.1	33.3	22.7	33.3
	31.3	34.3	23.4	30.6
STZ				
	24.1	24.4	23.9	24.8
	21.4	22.8	24.0	24.7
	25.6	20.3	24.7	22.4
	29.5	24.8	23.0	24.0
	23.3	20.4	23.5	24.3

### STZ-induced hyperglycemia preferentially affects mitochondria in the vMHb but not the LHb

**Figure 2:**
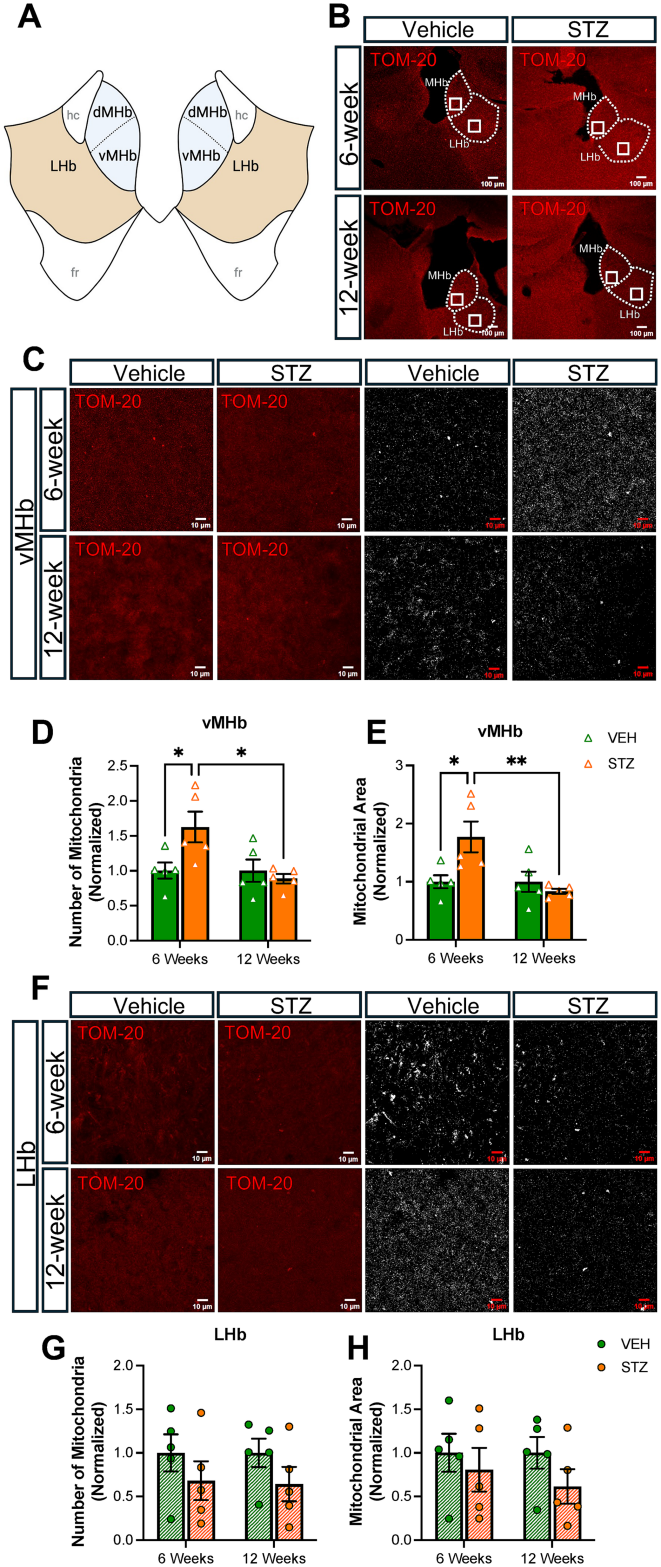
STZ-induced hyperglycemia increased mitochondrial numbers and altered morphology in vMHb but not LHb. **(A)** Diagram of the habenular complex. **(B)** Representative low-power (10X) confocal images of mitochondria (TOM-20) in the MHb and LHb for VEH and STZ groups at 6- and 12-week endpoints. **(C)** Representative vMHb high-power confocal images and mitochondria after processing (63x with zoom 1.0). Quantification of **(D)** the number of mitochondria and **(E)** mitochondrial area in the vMHb. **(F)** Representative LHb high-power confocal images and mitochondria after processing (63x with zoom 1.0). Quantification of **(G)** number of mitochondria and **(H)** mitochondrial area in the LHb. Images are maximum-intensity Z-projections derived from three to five Z-slices per mouse. Each data point represents the average per mouse. All statistical analyses were performed using two-way ANOVA followed by Tukey’s *post-hoc* test. All results are given as mean ± SEM (*p < 0.05, **p < 0.01). Created in https://BioRender.com.

Mitochondrial number and morphology are known to be dependent on the metabolic status of the cell (Chen et al., 2023), with high glucose concentration in the media decreasing *in vitro* mitochondrial biogenesis (Audzeyenka et al., 2021; Pahwa et al., 2020). In addition, hyperglycemia increased mitochondrial numbers in cardiomyocytes in rats (Kobayashi et al., 2020). Given that our STZ-treated mice have robust and chronic hyperglycemia, we analyzed mitochondrial number and distribution in the MHb with immunofluorescent staining for the outer mitochondrial membrane complex subunit 20 (TOM-20) at 6- and 12-week endpoints focusing our fields of view (FOV) on the ventral MHb (vMHb) where cholinergic neurons are present or the central LHb (Figures 2A,B). We did not examine the dorsal MHb in this study. Consistent with previous studies of mitochondria under hyperglycemic conditions, we found that the number of mitochondria was increased in the vMHb 6 weeks after STZ injection (number, normalized: VEH = 1.00 ± 0.25; STZ = 1.62 ± 0.49, *p* = 0.04; treatment *X* time: *F*_(1,16)_ = 6, *p* = 0.02; effect of treatment: *F*_(1,16)_ = 2.90, *p* = 0.1; effect of time: *F*_(1,16)_ = 6.01, *p* = 0.02). We found that the area occupied by mitochondria in each field of view, which reflects their intracellular distribution and fragmentation, was increased at the 6-week endpoint in the STZ group in the vMHb (%FOV, normalized: VEH = 1.00 ± 0.11; STZ = 1.77 ± 0.26, *p* = 0.02; treatment *X* time: *F*_(1,16)_ = 7.65, *p* = 0.01; effect of treatment: *F*_(1,16)_ = 3.22, *p* = 0.09; effect of time: *F*_(1,16)_ = 7.65, *p* = 0.01). Interestingly, we found that mitochondria were not different in number or occupied area between STZ and VEH groups at the 12-week endpoint (number, normalized: VEH = 1.00 ± 0.17; STZ = 0.83 ± 0.04) (Figures 2C–E), suggesting compensatory adaptation to sustained hyperglycemia. Consistent with a compensatory process, we found that the number of mitochondria in the vMHb at 6 weeks was significantly higher than the 12-week endpoint in the STZ-treated mice (STZ_6-week_ = 1.77 ± 0.26; STZ_12-week_ = 0.83 ± 0.04, *p* = 0.006). Given that the LHb has also been observed to play a role in metabolic response to insulin and to determine whether the observed effects of hyperglycemia were specific to the vMHb, which contains presumably vulnerable cholinergic neurons, we also examined mitochondria in the adjacent LHb. Neither mitochondrial distribution nor the number of mitochondria was significantly affected by hyperglycemia in the LHb (Figures 2F–H), although there was a strong trend for a decrease in the number of mitochondria in the STZ group at both 6- and 12-week endpoints (VEH_6-week_ = 1.00 ± 0.21, STZ_6-week_ = 0.68 ± 0.22, *p* = 0.67; VEH_12-week_ = 1.00 ± 0.16, STZ_12-week_ = 0.64 ± 0.19, *p* = 0.59; treatment *X* time: *F*_(1,16)_ = 0.009, *p* = 0.92; effect of treatment: *F*_(1,16)_ = 2.86, *p* = 0.1; effect of time: *F*_(1,16)_ = 0.009, *p* = 0.92). Together our findings suggest that the vMHb is more sensitive to chronic hyperglycemia in terms of mitochondrial number and distribution than the adjacent LHb, consistent with cholinergic vulnerability.

**Figure 3:**
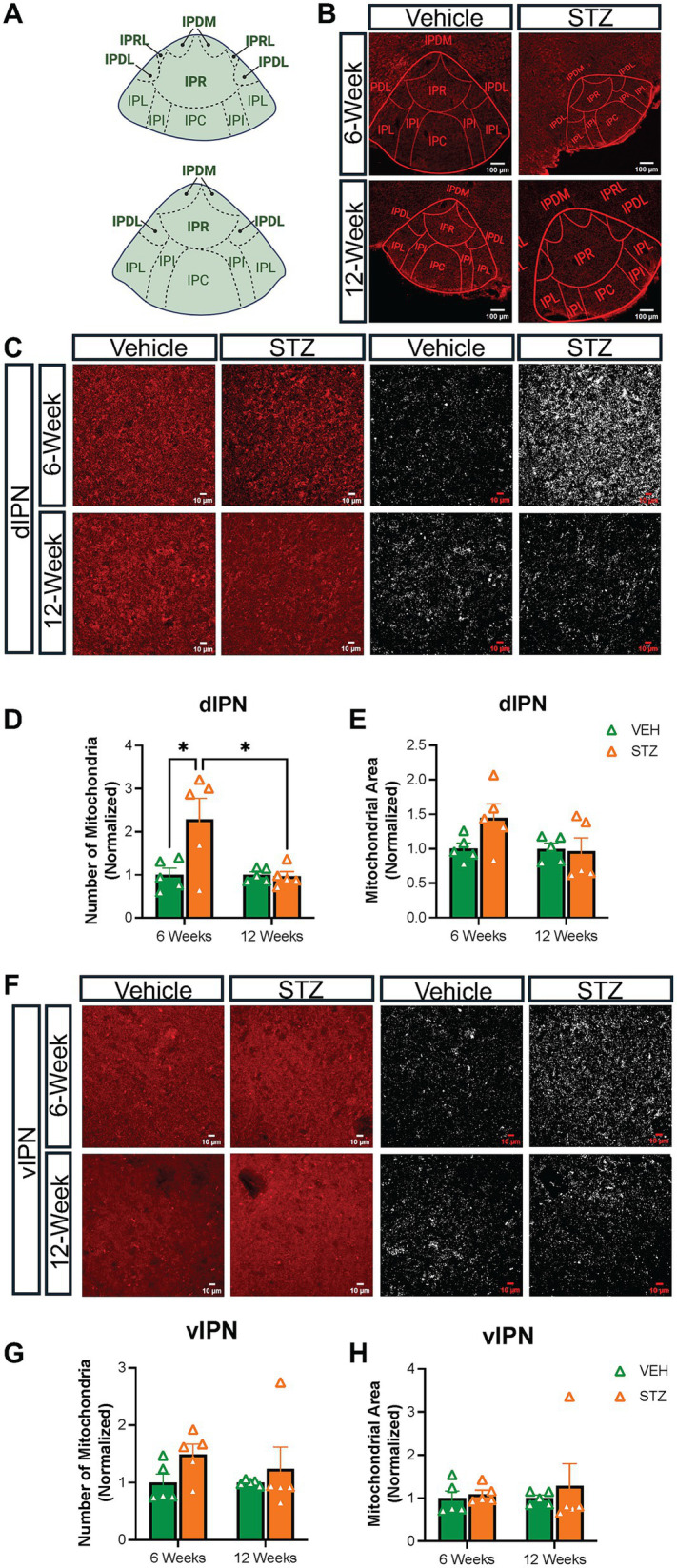
STZ-induced hyperglycemia predominantly affected mitochondria in the dIPN. **(A)** Diagram of representative sections of the IPN. Dorsal subnuclei are bolded. **(B)** Representative low-power confocal images of TOM-20 in the IPN for VEH and STZ groups at 6- and 12-week endpoints. **(C)** Representative dIPN high-power confocal images and mitochondria after processing (63x with zoom 1.0). Quantification of **(D)** number of mitochondria and **(E)** mitochondrial area in dIPN. **(F)** Representative vIPN high-power confocal images and mitochondria after processing (63x with zoom 1.0). Quantification of **(G)** number of mitochondria and **(H)** mitochondrial area in dIPN. Images are maximum intensity Z-projections derived from 3–5 Z-slices per mouse. Each data point represents the average of slices per mouse. All statistical analyses were performed using two-way ANOVA followed by Tukey’s *post-hoc* test. All results are given as mean ± SEM (*p < 0.05). Created in https://BioRender.com.

**Figure 4:**
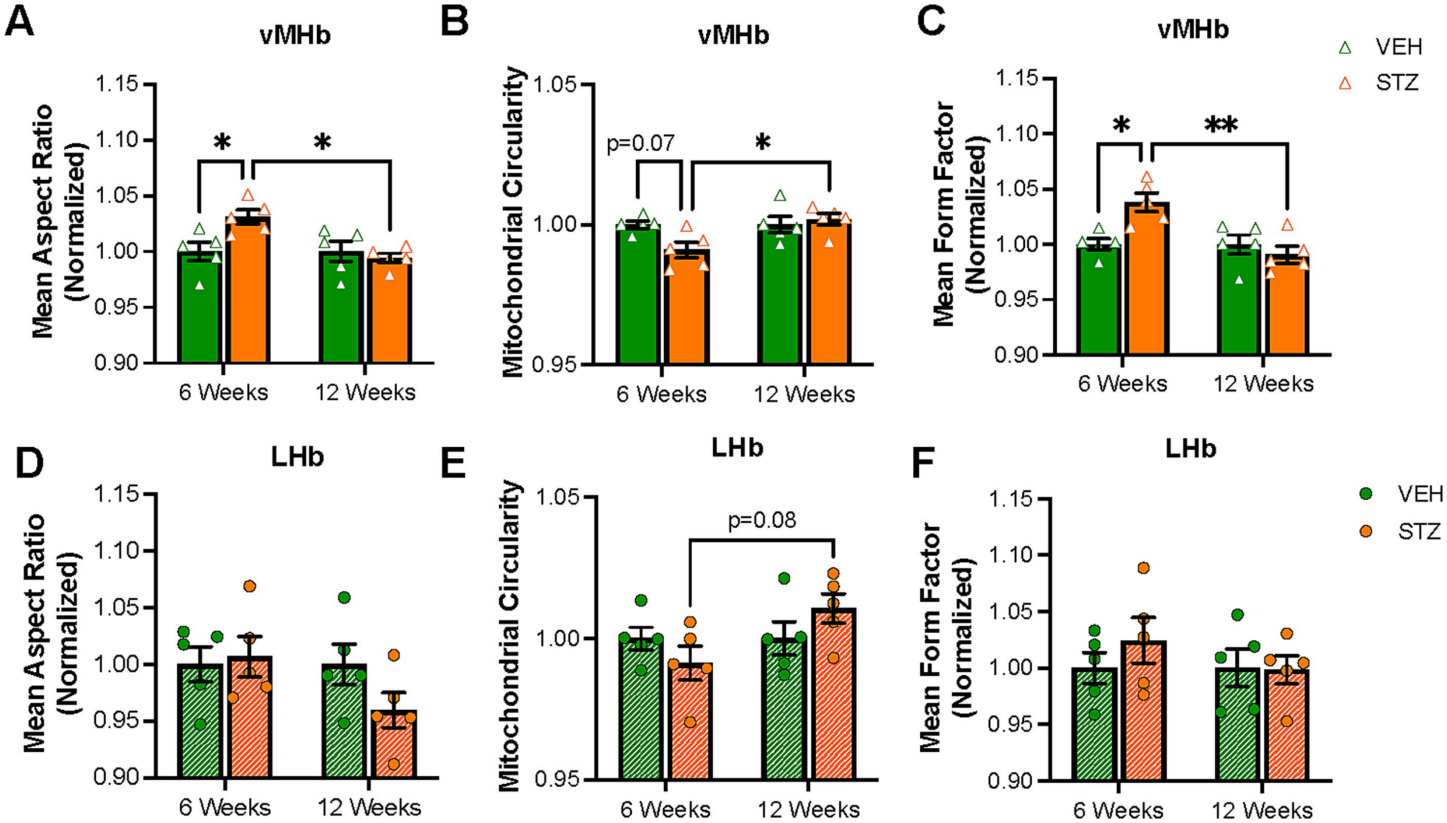
Mitochondrial elongation and network complexity are altered in the vMHb but not the LHb. Quantification of **(A)** mean aspect ratio (AR), **(B)** mitochondrial circularity, and **(C)** mean form factor (FF) in the MHb. Quantification of **(D)** mean AR, **(E)** mitochondrial circularity, and **(F)** mean FF in the LHb. Each data point represents the average per mouse. All statistical analyses were performed using two-way ANOVA followed by Tukey’s *post-hoc* test. All results are given as mean ± SEM (*p < 0.05, **p < 0.01).

Then, we utilized TOM-20 staining to evaluate mitochondrial morphology given that mitochondrial size and shape are elongated or fragmented depending on the conditions of the cell and that the shape of mitochondria has functional relevance. In the absence of an energy source, the mitochondrial aspect ratio (AR), which reflects the length-to-width ratio, is increased (Akinbiyi et al., 2021). Supplementary Figure S1 contains representative zoomed-in views of the processed images presented in Figures 2, 3. We found mitochondrial AR was increased significantly only at 6 weeks after STZ treatment in the vMHb (arbitrary units (AU): VEH_6-week_ = 1.00 ± 0.008; STZ_6-week_ = 1.03 ± 0.006, *p* = 0.04; VEH_12-week_ = 1.00 ± 0.009; STZ_12-week_ = 0.99 ± 0.004, *p* = 0.94; treatment *X* time: *F*_(1,16)_ = 6.3, *p* = 0.02; effect of treatment: *F*_(1,16)_ = 2.96, *p* = 0.1; effect of time: *F*_(1,16)_ = 6.30, *p* = 0.02). Like mitochondrial number, we found that the mean AR was significantly higher in 6- than 12-week STZ groups (*p* = 0.01) (Figure 4A, Table 2). Further analysis showed mitochondrial circularity, which is the opposite of AR, showed a strong trend to be decreased at the 6-week endpoint (dimensionless: VEH_6-week_ = 1.00 ± 0.001, STZ_6-week_ = 0.99 ± 0.003, *p* = 0.07; VEH_12-week_ = 1.00 ± 0.003, STZ_12-week_ = 1.002 ± 0.002, *p* = 0.94; treatment *X* time: *F*_(1,16)_ = 5.23, *p* = 0.04; effect of treatment: *F*_(1,16)_ = 2.20, *p* = 0.15; effect of time: *F*_(1,16)_ = 5.23, *p* = 0.04), which was consistent with increased AR. Again, mitochondrial circularity was significantly different between 6- and 12-week endpoints in STZ groups (*p* = 0.02) (Figure 4B). As was the case for mitochondrial distribution and numbers in LHb, we observed no significant effects of chronic hyperglycemia on AR (AU: VEH_6-week_ = 1.00 ± 0.02, STZ_6-week_ = 1.01 ± 0.02, *p* = 0.99; VEH_12-week_ = 1.00 ± 0.02, STZ_12-week_ = 0.96 ± 0.02, *p* = 0.35; treatment *X* time: *F*_(1,16)_ = 1.99, *p* = 0.18; effect of treatment: *F*_(1,16)_ = 0.99, *p* = 0.33; effect of time: *F*_(1,16)_ = 1.99, *p* = 0.18) although there was a trend for mitochondrial circularity (dimensionless: VEH_6-week_ = 1.00 ± 0.004, STZ_6-week_ = 0.99 ± 0.006, *p* = 0.66; VEH_12-week_ = 1.00 ± 0.006, STZ_12-week_ = 1.01 ± 0.005, *p* = 0.51; STZ_6-week_ vs. STZ_12-week_ *p* = 0.09; treatment *X* time: *F*_(1,16)_ = 3.28, *p* = 0.09; effect of treatment: *F*_(1,16)_ = 0.03, *p* = 0.86; effect of time: *F*_(1,16)_ = 3.28, *p* = 0.09) (Figures 4D,E).

**Table 2:** Raw values for mitochondrial analysis in vMHb with ImageJ.

Treatment	Mean aspect ratio (AU)	Mean form factor (AU)	Numbers (pixel unit)	Size (pixel^2^)	Area (%)	Integrated density (AU)	Circularity
Vehicle/6 week	1.787	1.363	10391	0.077	4.4667	19.728	0.924
	1.805	1.369	10640.667	0.077	4.598	19.772	0.924
	1.727	1.235	5691.5	0.055	1.707	13.822	0.959
	1.642	1.197	2652	0.059	0.826	15.102	0.967
	1.707	1.219	4242	0.052	1.210	13.318	0.963
STZ/6 week	1.822	1.435	11480	0.091	5.736	23.156	0.91
	1.779	1.291	9340	0.058	3.139	14.671	0.986
	1.727	1.246	5669	0.057	1.802	14.53	0.994
	1.743	1.236	5894	0.052	1.654	13.332	0.999
	1.757	1.265	8644.667	0.055	2.882	13.994	0.991
Vehicle/12 week	1.74	1.239	5387	0.049	1.463	12.581	0.962
	1.692	1.238	2543.5	0.048	0.667	12.251	0.975
	1.747	1.258	3556	0.057	1.132	14.652	0.965
	1.729	1.256	6249.5	0.055	1.964	13.895	0.958
	1.665	1.199	3548.333	0.053	1.060	13.666	0.964
STZ/ 12 week	1.723	1.231	4240.25	0.051	1.195	12.948	0.967
	1.705	1.219	3856	0.050	1.061	12.815	0.969
	1.703	1.216	4400	0.047	1.135	12.019	0.967
	1.713	1.206	3634.333	0.044	0.896	11.210	0.971
	1.680	1.260	2759.75	0.121	0.966	30.897	0.959

Mitochondria not only change their shape in response to the physiological condition of the cell (e.g., nutrient availability) but can also make complex networks to meet demands (Mitra et al., 2009). Form factor (FF), as defined by the perimeter^2^/(4π*area), represents the complexity of the mitochondrial network in the cell. Mitochondria tend to make branches and large networks to increase functional efficiency and impaired mitochondrial function is associated with more fragmented networks (Harmuth et al., 2018). Our results showed that chronic hyperglycemia significantly increased FF in the vMHb at the 6-week endpoint (AU: VEH_6-week_ = 1.00 ± 0.005, STZ_6-week_ = 1.03 ± 0.008, *p* = 0.01; VEH_12-week_ = 1.00 ± 0.009, STZ_12-week_ = 0.99 ± 0.008, *p* = 0.99; treatment *X* time: *F*_(1,16)_ = 10.03, *p* = 0.006; effect of treatment: *F*_(1,16)_ = 3.68, *p* = 0.07; effect of time: *F*_(1,16)_ = 10.03, *p* = 0.006). Again, we found that FF was significantly different between 6- and 12-week endpoints in vMHb of STZ mice (*p* = 0.001) (Figure 4C). Chronic hyperglycemia had no effects on mitochondrial network complexity in LHb at either 6- or 12-week endpoints (AU: VEH_6-week_ = 1.00 ± 0.014, STZ_6-week_ = 1.02 ± 0.020, *p* = 0.01; VEH_12-week_ = 1.00 ± 0.017, STZ_12-week_ = 0.99 ± 0.013, *p* = 0.99; treatment *X* time: *F*_(1,16)_ = 0.65, *p* = 0.43; effect of treatment: *F*_(1,16)_ = 0.65, *p* = 0.43; effect of time: *F*_(1,16)_ = 0.51, *p* = 0.49) (Figure 4F). Together, our data show that mitochondrial complexity and networking were influenced by chronic hyperglycemia only 6 weeks after STZ injection specifically in the vMHb.

**Figure 5:**
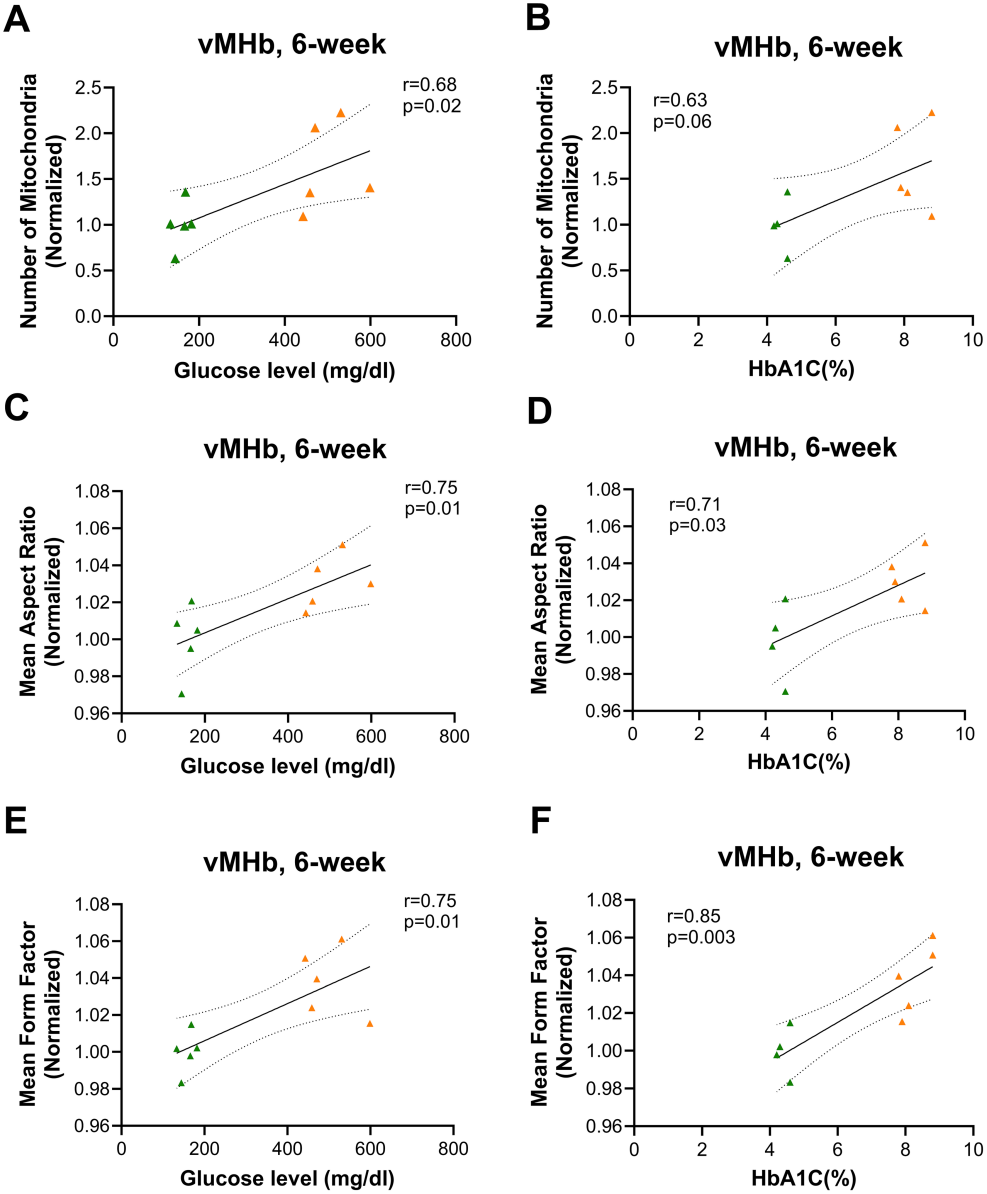
Mitochondrial distribution and morphology correlated with blood glucose and HbA1C (%) in vMHb at the 6-week endpoint. Mitochondrial numbers were correlated with **(A)** blood glucose and **(B)** HbA1C (%). The mean aspect ratio had a positive correlation with **(C)** blood glucose and **(D)** HbA1C (%). Mean form factor correlated with **(E)** blood glucose and **(F)** HbA1C (%). All statistical analyses were performed using the Pearson correlation coefficient followed by a two-tailed test and a 95% confidence interval.

To understand the relationship between glycemia and mitochondrial characteristics, we calculated the correlation between mitochondrial number and AR with blood glucose and HbA1C%. We found a positive correlation only at the 6-week time point between mitochondrial number and blood glucose and HbA1C in the vMHb (blood glucose: *F*_(1,8)_ = 7.25, R^2^ = 0.47, *p* = 0.02; HbA1C: *F*_(1,7)_ = 4.65, R^2^ = 0.39, *p* = 0.06) (Figures 5A,B). In addition, we found that mitochondrial shape and network complexity were significantly correlated with blood glucose and HbA1C in vMHb only at the 6-week endpoint (AR with blood glucose: *F*_(1,8)_ = 10.45, R^2^ = 0.56, *p* = 0.01; AR with HbA1C: *F*_(1,7)_ = 7.28, R^2^ = 0.50, *p* = 0.03; FF with blood glucose: *F*_(1,8)_ = 10.48, R^2^ = 0.56, *p* = 0.01, FF with HbA1C: *F*_(1,7)_ = 18.51, R^2^ = 0.72, *p* = 0.003) (Figures 5C–F).

Overall, we found that mitochondria were increased in number, elongated in shape, and formed more complex networks in the vMHb 6 weeks after the onset of hyperglycemia, largely consistent with an increase in available fuel, but the differences between VEH and STZ groups in the MHb did not persist out to 12 weeks after STZ injection. Our analysis showed that chronic hyperglycemia had no effects on mitochondrial number and complexity in the LHb, suggesting a specific effect on the vMHb. Consistent with a primary effect of available glucose as a fuel source, we found significant correlations between the mitochondrial network and numbers with blood glucose and HbA1C at the 6-week endpoint. These data suggest that mitochondria in the vMHb are most affected by hyperglycemia during the subacute stages of T1D and recover in later stages, perhaps due to compensatory changes.

### STZ-induced hyperglycemia differentially affects mitochondria in the dorsal and ventral IPN

The MHb projects topographically to the IPN, with the dorsal and ventral parts of the IPN (dIPN and vIPN) receiving projections primarily from cholinergic neurons in the vMHb, while the dorsal MHb sends substance P projections to the lateral IPN (Ables et al., 2023; Souter et al., 2022; Hsu et al., 2013). Given our interest in the effects on cholinergic vMHb neurons, we analyzed mitochondrial distribution and network at 6- and 12-week endpoints in the IPN nuclei that receive cholinergic MHb projections. Based on our observations, we grouped the IPN subnuclei into dorsal (dIPN: comprising the rostral, rostrolateral, dorsolateral, and dorsomedial subnuclei) and ventral (vIPN: comprising the intermediate and central subnuclei) divisions (Figure 3A). We excluded the lateral subnuclei from our analysis. In our previous study, we found that dIPN consists primarily of projection neurons to the raphe and laterodorsal tegmentum, while the vIPN consists of interneurons with connections within the IPN itself and receives reciprocal projections from the raphe (Ables et al., 2017). There are also distinctions in the types of synapses present in the dIPN versus vIPN, with the intermediate subnuclei of the vIPN characterized by a unique crest synapse of unknown significance from which mitochondria have been demonstrated to be excluded (Hamill and Lenn, 1983; Parajuli et al., 2020).

Consistent with the effects on the vMHb, we found that the number of mitochondria was significantly increased in dIPN at the 6- but not 12-week endpoint (number: VEH_6-week_: 1.00 ± 0.15, STZ_6-week_: 2.28 ± 0.48, *p* = 0.01; VEH_12-week_ = 1.00 ± 0.06, STZ_12-week_ = 0.96 ± 0.10, *p* = 0.99; STZ_6-week_ vs. STZ_12-week_, *p* = 0.01; treatment *X* time: *F*_(1,16)_ = 6.32, *p* = 0.02; effect of treatment: *F*_(1,16)_ = 5.68, *p* = 0.03; effect of time: *F*_(1,16)_ = 6.32, *p* = 0.02), while the occupied area was not significantly increased (%FOV: VEH_6-week_ = 1.00 ± 0.08; STZ_6-week_ = 1.45 ± 0.19, *p* = 0.2; VEH_12-week_ = 1.00 ± 0.10, STZ_12-week_ = 1.07 ± 0.19, *p* = 0.99; STZ_6-week_ vs. STZ_12-week_, *p* = 0.14; treatment *X* time: *F*_(1,16)_ = 2.61, *p* = 0.13; effect of treatment: *F*_(1,16)_ = 1.94, *p* = 0.18 and effect of time: *F*_(1,16)_ = 2.61, *p* = 0.13) (Figures 3C–E). In contrast, the vIPN did not demonstrate any significant changes in mitochondrial number (number: VEH_6-week_ = 1.00 ± 0.15, STZ_6-week_ = 1.49 ± 0.18, *p* = 0.43; VEH_12-week_ = 1.00 ± 0.02, STZ_12-week_ = 1.23 ± 0.38, *p* = 0.87; STZ_6-week_ vs. STZ_12-week_, *p* = 0.85; treatment *X* time: *F*_(1,16)_ = 0.31, *p* = 0.58; effect of treatment: *F*_(1,16)_ = 2.65, *p* = 0.21; effect of time: *F*_(1,16)_ = 0.31, *p* = 0.58) or occupied area (%FOV: VEH_6-week_ = 1.00 ± 0.16, STZ_6-week_ = 1.09 ± 0.09, *p* = 0.99; VEH_12-week_ = 1.00 ± 0.06, STZ_12-week_ = 1.28 ± 0.51, *p* = 0.88; STZ_6-week_ vs. STZ_12-week_, *p* = 0.82; treatment *X* time: *F*_(1,16)_ = 0.12, *p* = 0.74; effect of treatment: *F*_(1,16)_ = 0.45, *p* = 0.51; effect of time: *F*_(1,16)_ = 0.12, *p* = 0.74), although there was a strong trend for higher mitochondrial number at the 6-week endpoint (Figures 3F–H; Tables 3, 4).

**Table 3:** Raw values for mitochondrial analysis in dIPN with ImageJ.

Treatment	Mean aspect ratio (AU)	Mean form factor (AU)	Numbers (pixel unit)	Size (pixel^2^)	Area (%)	Integrated density (AU)	Circularity
Vehicle/6 Week	1.93	1.41	5139	0.163	1.829	41.582	0.953
	2.005	1.499	7222.333	0.151	2.407	38.543	0.95
	1.876	1.134	4211.333	0.160	1.502	40.838	0.956
	1.911	1.139	6082.667	0.666	8.587	169.957	0.88
	1.865	1.121	2617.333	1.263	7.281	321.984	0.832
STZ/6 Week	1.914	1.367	9399	0.181	3.959	46.142	0.937
	1.918	1.4	3595	0.213	1.607	54.171	0.951
	1.919	1.435	13091.333	0.412	11.375	104.976	0.878
	1.909	1.180	12492.667	0.379	10.441	96.689	0.887
	1.838	1.134	13964	0.407	12.611	103.935	0.873
Vehicle/12 Week	1.899	1.289	5875	0.277	3.765	70.729	0.926
	1.846	1.126	7895	0.308	5.432	78.515	0.914
	1.796	1.135	2681.667	0.183	1.05	46.694	0.953
	1.846	1.118	2221.333	0.252	1.186	64.255	0.947
	#	#	1965	0.188	0.819	47.933	0.956
STZ/12 Week	1.907	1.218	5981.667	0.217	2.863	55.308	0.931
	1.854	1.2085	4936.5	0.265	2.996	67.494	0.931
	1.902	1.198	6733	0.211	3.189	53.747	0.932
	1.801	1.142	6188.25	0.455	6.381	115.955	0.911
	2.709	1.323	3122.667	0.233	1.504	59.446	0.945

#: Bubble obscured analysis.

**Table 4:** Raw values for mitochondrial analysis in vIPN with ImageJ.

Treatment	Mean aspect ratio (AU)	Mean form factor (AU)	Numbers (pixel unit)	Size (pixel^2^)	Area (%)	Integrated density (AU)	Circularity
Vehicle/6 week	1.926	1.312	3560.333	0.191	1.146	48.571	0.96
	1.956	1.48	3728	0.132	1.089	33.79	0.965
	1.839	1.104	7035	0.151	2.362	38.529	0.95
	1.902	1.102	7639.667	0.316	5.419	80.464	0.904
	1.924	1.146	4691	0.857	8.794	218.593	0.855
STZ/6 week	1.908	1.291	6557.333	0.15	2.186	38.17	0.953
	1.893	1.289	4110.667	0.218	1.781	55.564	0.945
	1.911	1.285	10011	0.308	6.983	78.713	0.903
	1.914	1.135	10299	0.287	6.573	73.184	0.903
	1.842	1.145	11880.106	0.257	6.825	65.6	0.904
Vehicle/12 week	1.909	1.885333333	7784	0.214	3.6963	54.464	0.929
	1.844	1.865	7436.333	0.279	5.057	71.041	0.922
	1.799	1.908	1873.333	0.186	0.677	47.401	0.96
	1.857	1.866	1998.333	0.208	0.913	53.063	0.953
	#	1.907	2127	0.169	0.787	43.157	0.963
STZ/12 week	1.885333333	1.208	7033.667	0.216	3.365	55.104	0.931
	1.86475	1.197	5000.5	0.251	2.886	63.841	0.936
	1.908	1.225	7156.75	0.216	3.54	55.161	0.928
	1.865666667	1.201	7042.75	0.227	3.574	57.771	0.935
	1.9065	1.212	5496.667	0.216	2.657	55.07	0.931

#: Bubble obscured analysis.

**Figure 6:**
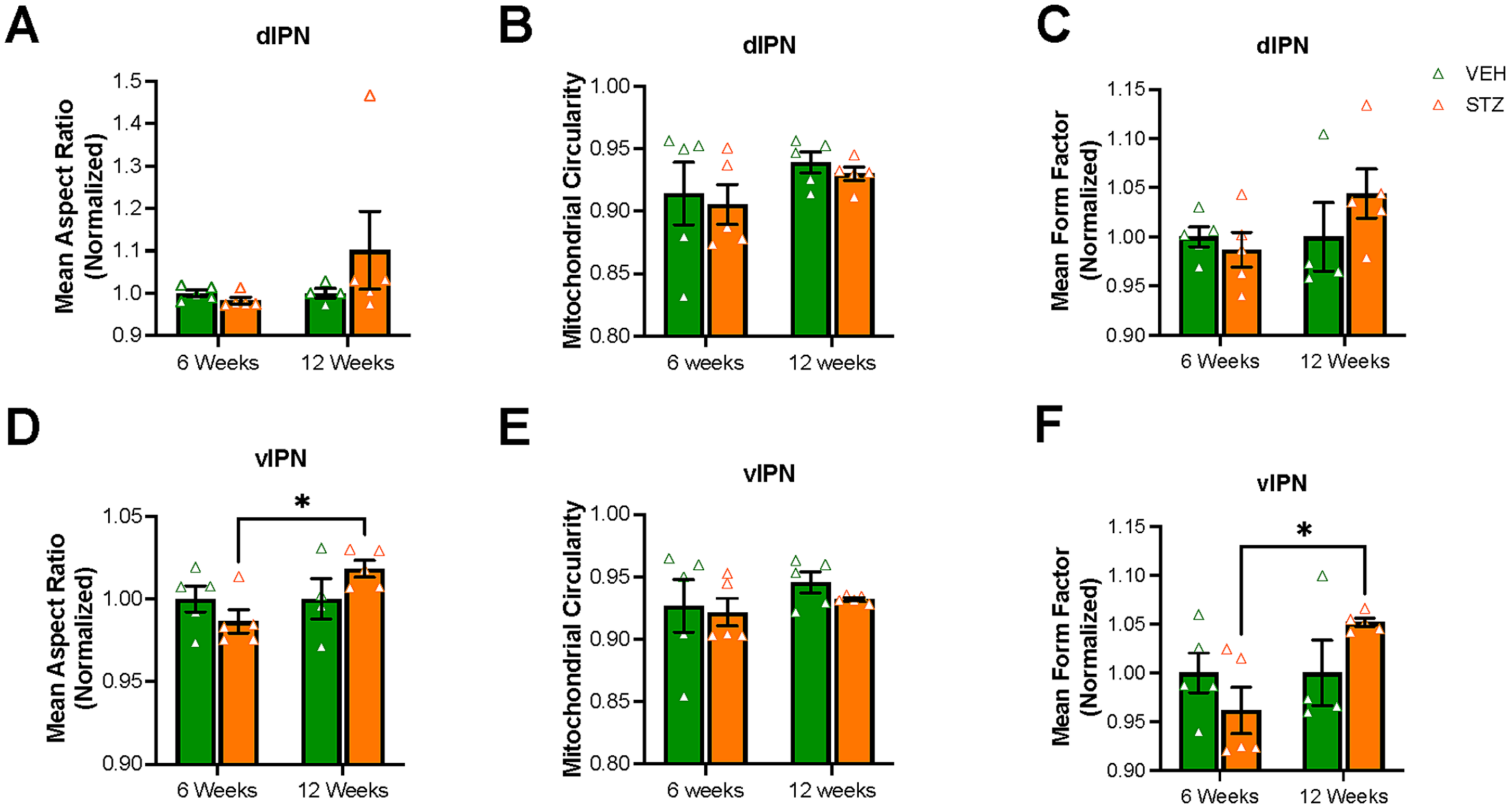
Mitochondrial elongation and network complexity are altered in the vIPN but not the dIPN. Quantification of **(A)** mean aspect ratio (AR), **(B)** mitochondrial circularity and **(C)** mean form factor (FF) in the dIPN. Quantification of **(D)** mean AR, **(E)** mitochondrial circularity, and **(F)** mean FF in the vIPN. Each data point represents the average per mouse. All statistical analyses were performed using two-way ANOVA followed by Tukey’s *post-hoc* test. All results are given as mean ± SEM (*p < 0.05).

We next examined the AR, circularity, and FF of mitochondria in the IPN. When examining the morphology of mitochondria within the dIPN, mean AR values were not significantly changed at any endpoint (AU: VEH_6-week_ = 1.00 ± 0.007, STZ_6-week_ = 0.98 ± 0.008, *p* = 0.99; VEH_12-week_ = 1.00 ± 0.01, STZ_12-week_ = 1.10 ± 0.09, *p* = 0.51; STZ_6-week_ vs. STZ_12-week_, *p* = 0.32; treatment *X* time: *F*_(1,15)_ = 1.45, *p* = 0.25; effect of treatment: *F*_(1,15)_ = 0.72, *p* = 0.40; effect of time: *F*_(1,15)_ = 1.45, *p* = 0.25), nor was circularity (dimensionless: VEH_6-week_ = 0.914 ± 0.025, STZ_6-week_ = 0.905 ± 0.016, *p* = 0.98; VEH_12-week_ = 0.939 ± 0.008, STZ_12-week_ = 0.930 ± 0.005, *p* = 0.98; STZ_6-week_ vs. STZ_12-week_, *p* = 0.69; treatment *X* time: *F*_(1,16)_ = 0.00004, *p* = 0.99; effect of treatment: *F*_(1,16)_ = 0.32, *p* = 0.58; effect of time: *F*_(1,16)_ = 2.50, *p* = 0.13) (Figures 6A,B). Network analysis of mitochondria showed no change in dIPN at the 6- or 12-week endpoints as measured by mean FF (AU: VEH_6-week_ = 1.00 ± 0.01, STZ_6-week_ = 0.98 ± 0.01, *p* = 0.97; VEH_12-week_ = 1.00 ± 0.03, STZ_12-week_ = 1.04 ± 0.02, *p* = 0.54; STZ_6-week_ vs. STZ_12-week_, *p* = 0.29; treatment *X* time: *F*_(1,15)_ = 1.61, *p* = 0.22; effect of treatment: *F*_(1,15)_ = 0.48, *p* = 0.50; effect of time: *F*_(1,15)_ = 1.61, *p* = 0.22) (Figure 6C). Despite no change in number, mitochondrial network analysis in vIPN found that mean AR (AU: VEH_6-week_ = 1.00 ± 0.008, STZ_6-week_ = 0.98 ± 0.007, *p* = 0.61; VEH_12-week_ = 1.00 ± 0.01, STZ_12-week_ = 1.01 ± 0.005, *p* = 0.42; STZ_6-week_ vs. STZ_12-week_, *p* = 0.05; treatment *X* time: *F*_(1,15)_ = 3.94, *p* = 0.07; effect of treatment: *F*_(1,15)_ = 0.09, *p* = 0.77; effect of time: *F*_(1,15)_ = 3.94, *p* = 0.07) and mean FF (AU: VEH_6-week_ = 1.00 ± 0.02, STZ_6-week_ = 0.96 ± 0.02, *p* = 0.58; VEH_12-week_ = 1.00 ± 0.03, STZ_12-week_ = 1.05 ± 0.004, *p* = 0.38; STZ_6-week_ vs. STZ_12-week_, *p* = 0.04; treatment *X* time: *F*_(1,15)_ = 4.32, *p* = 0.06; effect of treatment: *F*_(1,15)_ = 0.10, *p* = 0.75; effect of time: *F*_(1,15)_ = 4.32, *p* = 0.06) were significantly different between 6- and 12-week endpoints, while circularity was not significantly affected (dimensionless: VEH_6-week_ = 0.93 ± 0.02, STZ_6-week_ = 0.92 ± 0.01, *p* = 0.99; VEH_12-week_ = 0.95 ± 0.01, STZ_12-week_ = 0.93 ± 0.001, *p* = 0.87; STZ_6-week_ vs. STZ_12-week_, *p* = 0.93; treatment *X* time: *F*_(1,16)_ = 0.11, *p* = 0.74; effect of treatment: *F*_(1,16)_ = 0.54, *p* = 0.47; effect of time: *F*_(1,16)_ = 1.36, *p* = 0.26) (Figures 6D–F).

**Figure 7:**
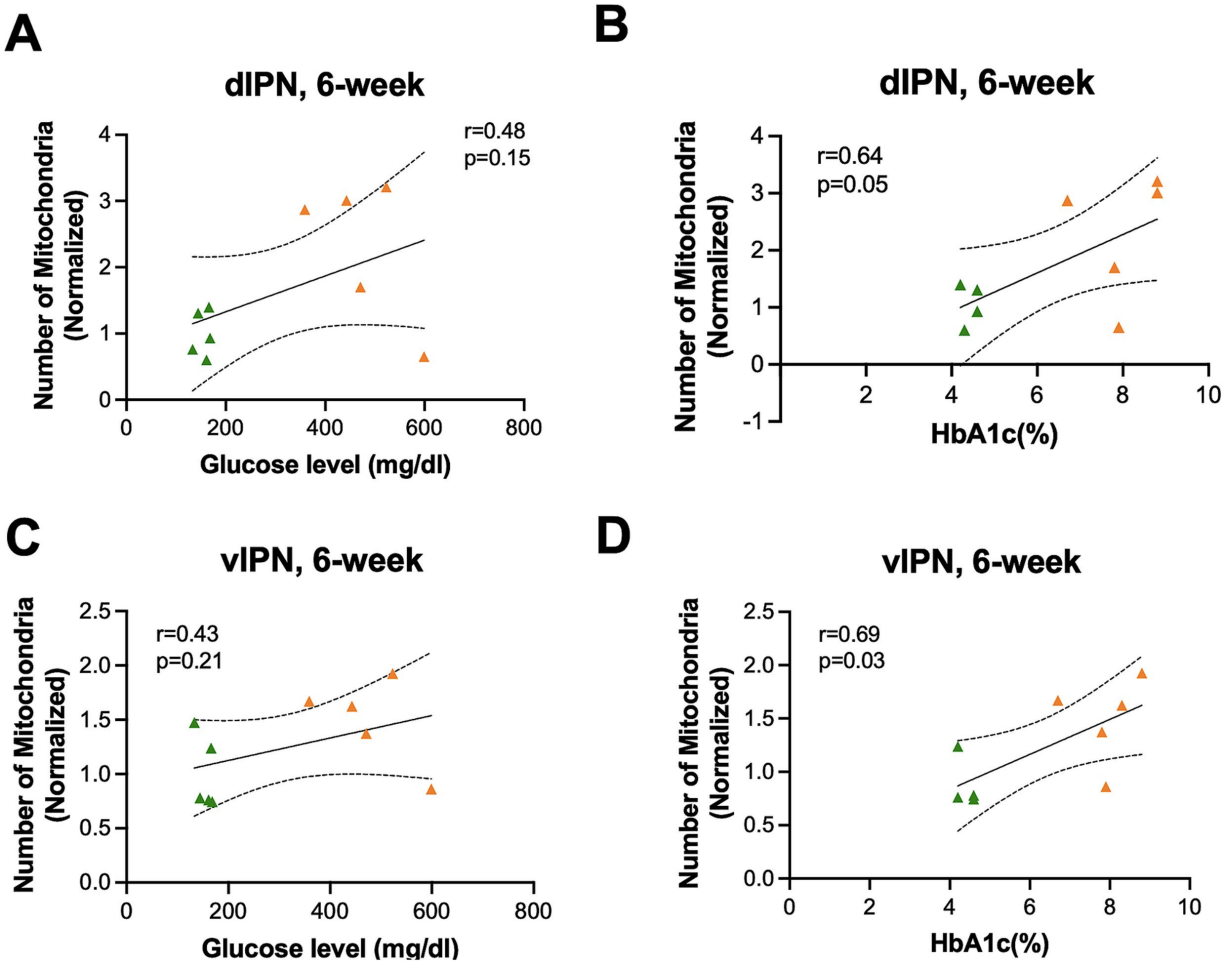
Mitochondrial distribution and morphology correlated with HbA1C (%) but not blood glucose in the IPN at the 6-week endpoint. Mitochondrial numbers were correlated with **(A)** blood glucose and **(B)** HbA1C (%) in the dIPN. Mitochondrial numbers were correlated with **(C)** blood glucose and **(D)** HbA1C (%) in the vIPN. All statistical analyses were performed using the Pearson correlation coefficient followed by a two-tailed test and a 95% confidence interval.

We again evaluated the relationship between mitochondrial distribution with blood glucose and HbA1C at 6-week endpoint in both dIPN (mitochondrial number with blood glucose: *F*_(1,8)_ = 2.40, R^2^ = 0.23, *p* = 0.15 and HbA1C: *F*_(1,7)_ = 5.05, R^2^ = 0.41, *p* = 0.05) (Figures 7A,B) and vIPN (mitochondrial number with blood glucose: *F*_(1,8)_ = 1.82, R^2^ = 0.18, *p* = 0.21 and HbA1C: *F*_(1,7)_ = 6.63, R^2^ = 0.48, *p* = 0.03) (Figures 7C,D) and found that unlike the vMHb, where both acute and chronic measures of glycemic control correlated with mitochondrial dynamics, the IPN appears to respond to only chronic measures of glycemic control. Together, our results demonstrated that mitochondrial dynamics were differentially affected in the dIPN compared to the vIPN, with mitochondrial number significantly increased in the dIPN at 6-week endpoint while the vIPN showed a stronger response to chronic hyperglycemia in terms of mitochondrial network.

### STZ-induced hyperglycemia influences lipid accumulation differently in the MHb, IPN, and ventral tegmental area (VTA)

**Figure 8:**
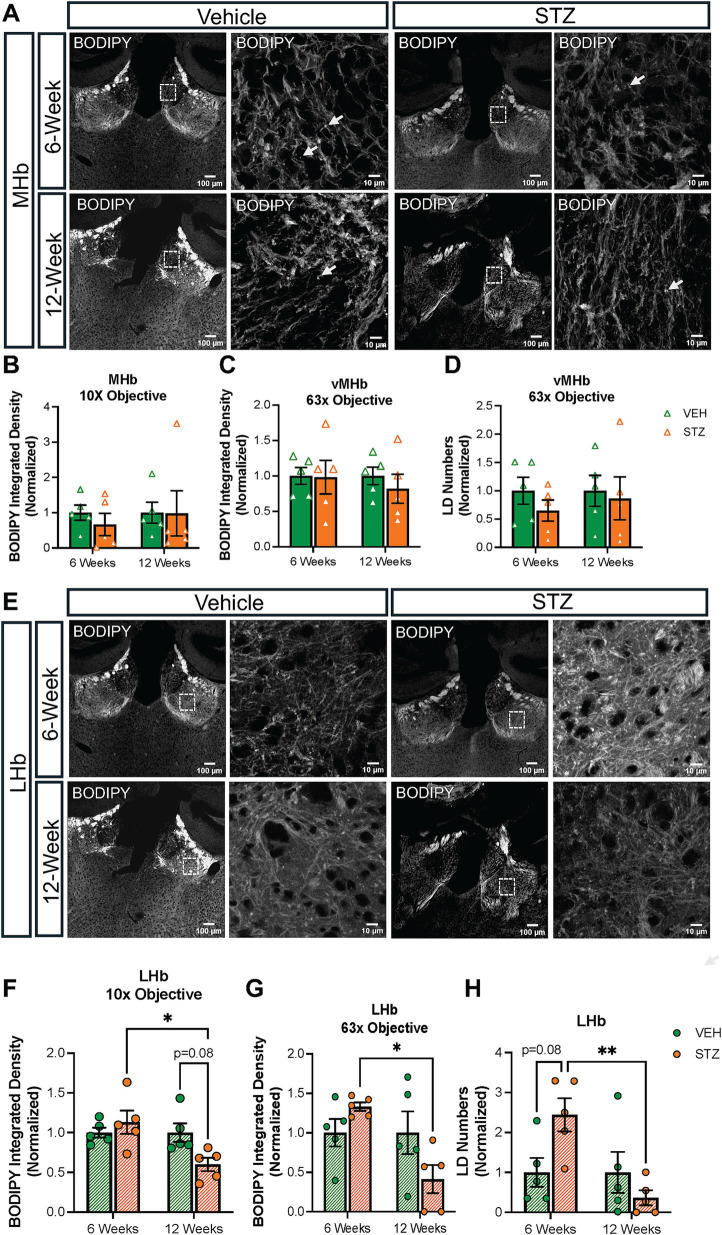
STZ-induced hyperglycemia influenced lipid formation in the LHb but not the MHb. **(A)** Representative confocal images of BODIPY^493/503^ in MHb with low-power (10X) and high-power (63X) objectives. Quantification of BODIPY^493/503^ in **(B)** MHb with low-power (10X) and **(C)** in vMHb with high-power (63X) objectives and of **(D)** LD numbers in the vMHb (63x objective). **(E)** Representative confocal images of BODIPY^493/503^ in LHb with low-power (10X) and high-power (63X) objectives. Quantification of BODIPY^493/503^ in **(F)** LHb with low-power (10X) and **(G)** in LHb with high-power (63X) objectives and of **(H)** LD numbers in the LHb (63x objective). Images are maximum intensity Z-projections derived from 3–5 Z-slices per mouse. Each data point represents the average of slices per mouse. All statistical analyses were performed using two-way ANOVA followed by Tukey’s *post-hoc* test. All results are given as mean ± SEM (*p < 0.05, **p < 0.01).

Lipid droplet (LD) formation within cells is associated with cellular energy status, with excess glucose being converted to fatty acid and stored within LDs. Conversely, when glucose levels are low, mitochondria can utilize lipids to generate energy through *β*-oxidation. After finding a difference in mitochondrial distribution and network in the vMHb and IPN of mice with chronic hyperglycemia, we analyzed LDs in these regions using BODIPY^493/503^ to probe neutral lipids (Figure 8A). Integrated density of BODIPY^493/503^ was not significantly affected at 6- and 12-week endpoints in the MHb (AU: VEH_6-week_ = 1.00 ± 0.21, STZ_6-week_ = 0.66 ± 0.31, *p* = 0.76; VEH_12-week_ = 1.00 ± 0.29, STZ_12-week_ = 0.98 ± 0.64, *p* = 0.23; STZ_6-week_ vs. STZ_12-week_, *p* = 0.74; treatment *X* time: *F*_(1,16)_ = 0.51, *p* = 0.49; effect of treatment: *F*_(1,16)_ = 4.4, *p* = 0.05; effect of time: *F*_(1,16)_ = 0.51, *p* = 0.48) (Figure 8B). Moreover, when we limited our analysis to the vMHb, we still found no significant differences in integrated density (AU: VEH_6-week_ = 1.00 ± 0.11, STZ_6-week_ = 0.98 ± 0.23, *p* = 0.99; VEH_12-week_ = 1.00 ± 0.12, STZ_12-week_ = 0.81 ± 0.20, *p* = 0.88; STZ_6-week_ vs. STZ_12-week_, *p* = 0.91; treatment *X* time: *F*_(1,16)_ = 0.30, *p* = 0.58; effect of treatment: *F*_(1,16)_ = 0.30, *p* = 0.58; effect of time: *F*_(1,16)_ = 0.21, *p* = 0.65) (Figure 8C). Similarly, we found that LD number in the vMHb was not significantly reduced at either 6- or 12-week endpoints (number: VEH_6-week_ = 1.00 ± 0.23, STZ_6-week_ = 0.64 ± 0.18, *p* = 0.80; VEH_12-week_ = 1.00 ± 0.27, STZ_12-week_ = 0.86 ± 0.37, *p* = 0.98; STZ_6-week_ vs. STZ_12-week_, *p* = 0.94; treatment *X* time: *F*_(1,16)_ = 0.76, *p* = 0.39; effect of treatment: *F*_(1,16)_ = 0.76, *p* = 0.39; effect of time: *F*_(1,16)_ = 0.15, *p* = 0.70) (Figure 8D).

Surprisingly, analysis of the LHb (Figure 8E) revealed that BODIPY^493/503^ integrated density had distinct temporal dynamics in response to chronic hyperglycemia at the different endpoints (AU: VEH_6-week_ = 1.00 ± 0.06, STZ_6-week_ = 1.13 ± 0.14, *p* = 0.82; VEH_12-week_ = 1.00 ± 0.11, STZ_12-week_ = 0.60 ± 0.08, *p* = 0.08; STZ_6-week_ vs. STZ_12-week_, *p* = 0.01; treatment *X* time: *F*_(1,16)_ = 6.11, *p* = 0.03; effect of treatment: *F*_(1,16)_ = 1.57, *p* = 0.22; effect of time: *F*_(1,16)_ = 6.11, *p* = 0.03) despite no changes in mitochondrial measures (Figure 8F). By focusing on the central part of the LHb, we found that both LD numbers and integrated density decreased significantly at the 12-week endpoint in comparison to the 6-week endpoint in the STZ group (LD numbers: VEH_6-week_ = 1.00 ± 0.36, STZ_6-week_ = 2.44 ± 0.41, *p* = 0.08; VEH_12-week_ = 1.00 ± 0.51, STZ_12-week_ = 0.36 ± 0.19, *p* = 0.65; STZ_6-week_ vs. STZ_12-week_, *p* = 0.007; treatment *X* time: *F*_(1,16)_ = 7.17, *p* = 0.02; effect of treatment: *F*_(1,16)_ = 1.07, *p* = 0.31; effect of time: *F*_(1,16)_ = 7.17, *p* = 0.02; BODIPY^493/503^ integrated density (AU): VEH_6-week_ = 1.00 ± 0.17, STZ_6-week_ = 1.33 ± 0.05, *p* = 0.60; VEH_12-week_ = 1.00 ± 0.27, STZ_12-week_ = 0.41 ± 0.17, *p* = 0.15; STZ_6-week_ vs. STZ_12-week_, *p* = 0.01; treatment *X* time: *F*_(1,16)_ = 6.10, *p* = 0.03; effect of treatment: *F*_(1,16)_ = 0.47, *p* = 0.49; effect of time: *F*_(1,16)_ = 6.10, *p* = 0.03) (Figures 8G,H).

**Figure 9:**
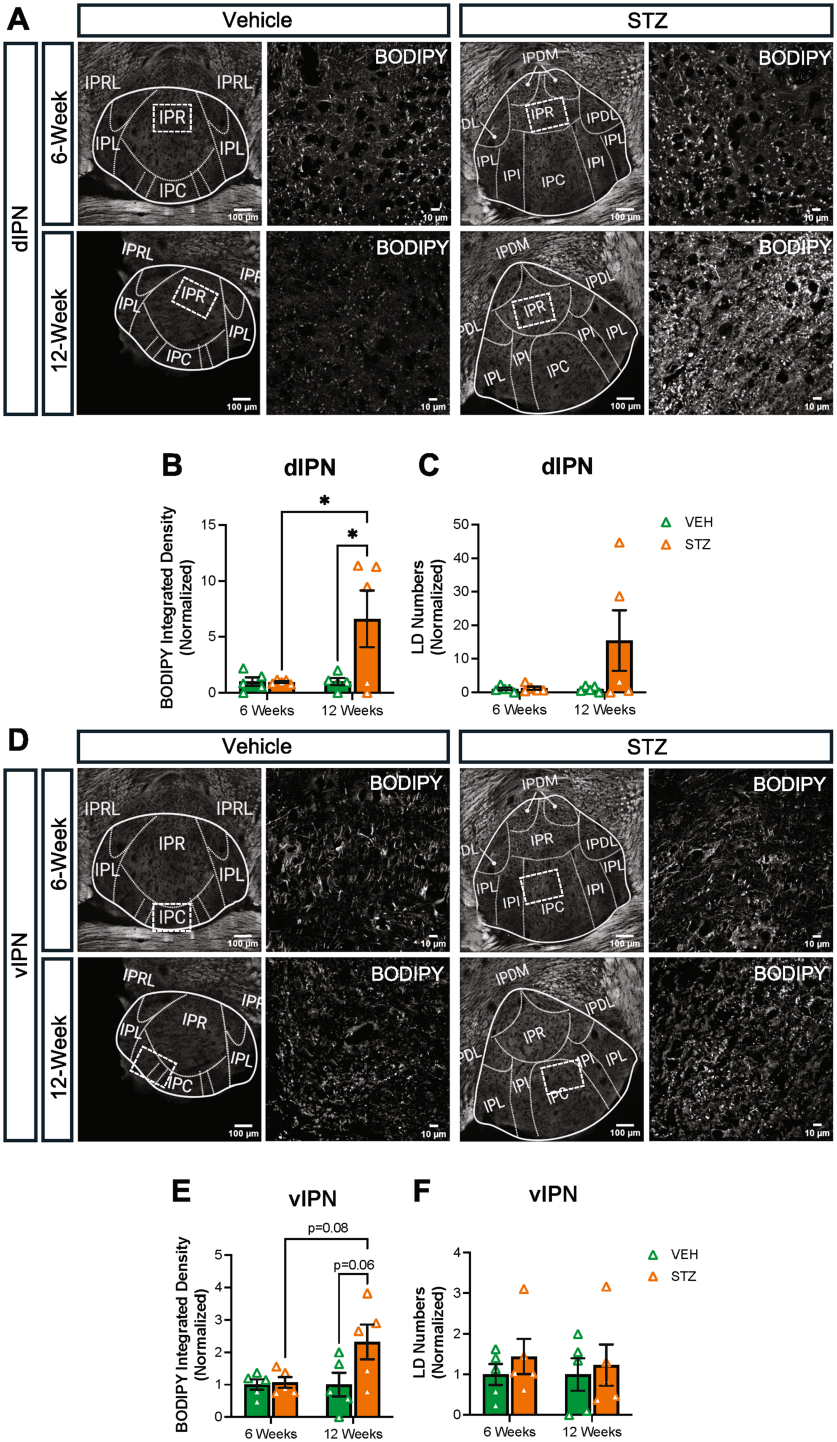
STZ-induced hyperglycemia increased lipid formation in IPN at 12 weeks. **(A)** Representative confocal images of BODIPY^493/503^ in dIPN. Quantification of **(B)** integrated density of BODIPY^493/503^ in dIPN and **(C)** LD numbers in dIPN. **(D)** Representative confocal images of BODIPY^493/503^ in vIPN. Quantification of **(E)** integrated density of BODIPY^493/503^ in vIPN and **(F)** LD numbers in vIPN. Images are maximum-intensity Z-projections derived from three to five Z-slices per mouse. Each data point represents the average of slices per mouse. All statistical analyses were performed using two-way ANOVA followed by Tukey’s *post-hoc* test. All results are given as mean ± SEM (*p < 0.05).

Then, we checked BODIPY^493/503^ integrated density and LD numbers in both dIPN and vIPN (Figure 9). Our analysis showed integrated density was increased in dIPN at the 12-week endpoint in the STZ group. Moreover, STZ groups at 6- and 12-week endpoints were significantly different (VEH_6-week_ = 1.00 ± 0.37, STZ_6-week_ = 0.96 ± 0.08, *p* = 0.99; VEH_12-week_ = 1.00 ± 0.31, STZ_12-week_ = 6.60 ± 2.53, *p* = 0.03; STZ_6-week_ vs. STZ_12-week_, *p* = 0.03; treatment *X* time: *F*_(1,16)_ = 4.75, *p* = 0.04; effect of treatment: *F*_(1,16)_ = 4.62, *p* = 0.04; effect of time: *F*_(1,16)_ = 4.75, *p* = 0.04) (Figure 9B). The number of LDs was unchanged at the 6-week endpoint (*p* > 0.99) and increased but not significantly at the 12-week endpoint in the STZ group (*p* = 0.15) (Figure 9C). Analysis of the vIPN showed a strong trend (*p* = 0.065) for increased lipid integrated density at the 12-week endpoint in the STZ group, while STZ treatment had no effect on BODIPY^493/503^ integrated density at the 6-week endpoint (AU: VEH_6-week_ = 1.00 ± 0.15, STZ_6-week_ = 1.06 ± 0.16, *p* = 0.99; VEH_12-week_ = 1.00 ± 0.36, STZ_12-week_ = 2.32 ± 0.53, *p* = 0.065; STZ_6-week_ vs. STZ_12-week_, *p* = 0.085; treatment *X* time: *F*_(1,16)_ = 3.32, *p* = 0.087; effect of treatment: *F*_(1,16)_ = 4.09, *p* = 0.06; effect of time: *F*_(1,16)_ = 3.32, *p* = 0.087) (Figure 9E). We found no significant difference in the number of LDs at either 6- or 12-week endpoints in vIPN (VEH_6-week_ = 1.00 ± 0.25, STZ_6-week_ = 1.44 ± 0.43, *p* = 0.86; VEH_12-week_ = 1.00 ± 0.40, STZ_12-week_ = 1.23 ± 0.51, *p* = 0.97; STZ_6-week_ vs. STZ_12-week_, *p* = 0.98; treatment *X* time: *F*_(1,16)_ = 3.32, *p* = 0.087; effect of treatment: *F*_(1,16)_ = 4.08, *p* = 0.06; effect of time: *F*_(1,16)_ = 3.32, *p* = 0.087) (Figure 9F). Overall, our findings suggest that lipid accumulation in the IPN is increased in the STZ group at the 12-week endpoint, with a stronger effect on the dIPN. These findings suggest that STZ-induced hyperglycemia differentially affects lipid accumulation in the MHb, LHb, and IPN, with the IPN demonstrating increased lipid accumulation at later stages.

**Figure 10:**
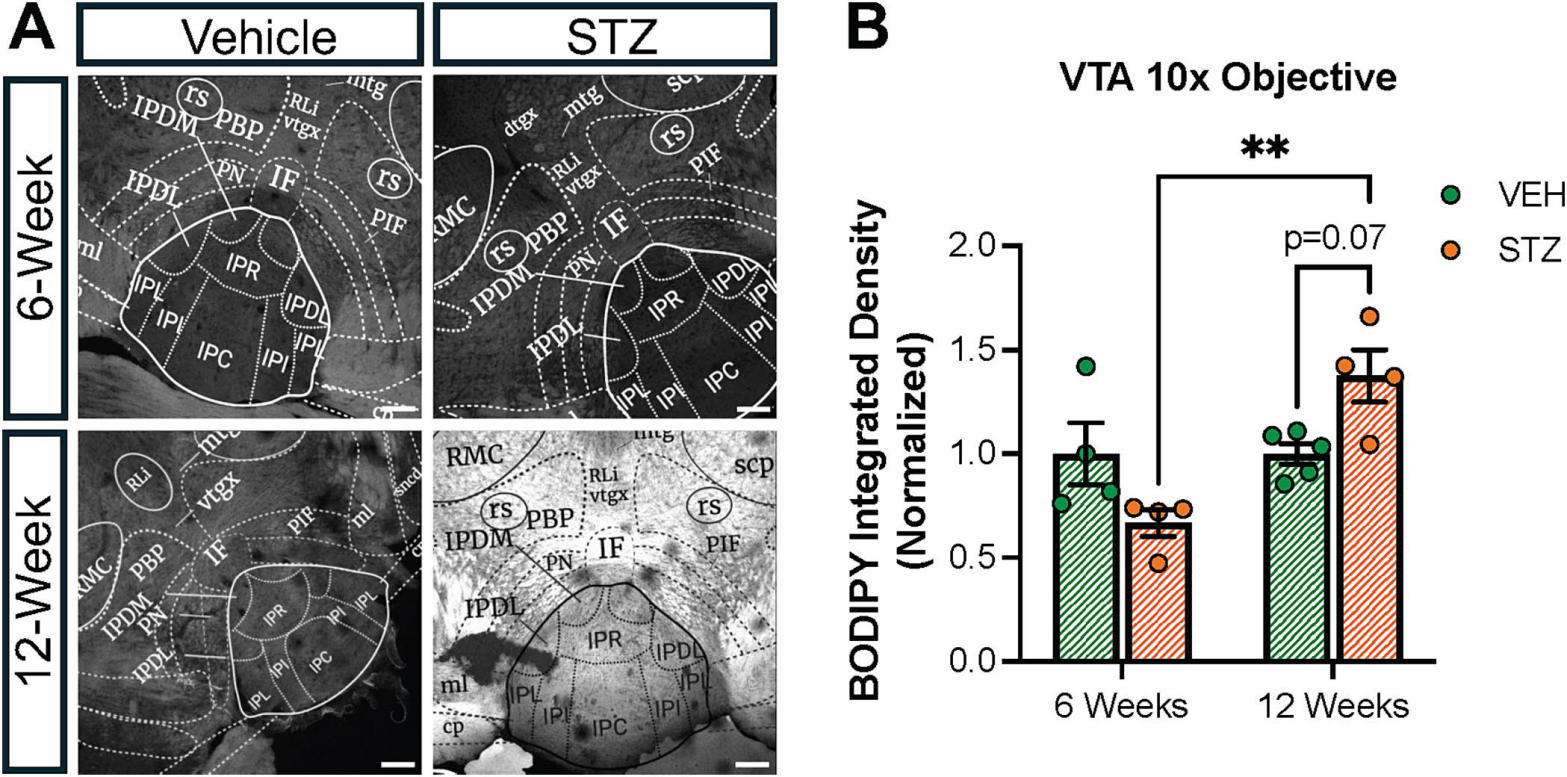
STZ-induced hyperglycemia altered lipid formation dynamics in the VTA. **(A)** Representative confocal images of BODIPY^493/503^ in VTA. Quantification of **(B)** Integrated density of BODIPY^493/503^ in the interfascicular nucleus (IF) of VTA. Images are maximum intensity Z-projections derived from 3–5 Z-slices per mouse. Each data point represents the average of slices per mouse. All statistical analyses were performed using two-way ANOVA followed by Tukey’s *post-hoc* test. All results are given as mean ± SEM (*p < 0.05, **p < 0.01).

The ventral tegmental area (VTA) has a crucial role in the reward system in the brain, providing the source of dopamine to signal reward in the nucleus accumbens. Recently, it was shown that the VTA has inputs to IPN in the mouse (Molas et al., 2023). In addition, nicotine withdrawal-induced anxiety behavior through VTA-IPN-MHb in male mice (Zhao-Shea et al., 2015). Given the role of the VTA in reward and the link between diabetes and mood disorders, we checked the lipid density in the VTA at the 6- and 12-week endpoints. We found that BODIPY^493/503^ integrated density decreased slightly at the 6-week endpoint in the VTA (VEH_6-week_ = 1.00 ± 0.14, STZ_6-week_ = 0.66 ± 0.06, *p* = 0.15; VEH_12-week_ = 1.00 ± 0.04, STZ_12-week_ = 1.37 ± 0.12, *p* = 0.076; STZ_6-week_ vs. STZ_12-week_, *p* = 0.001; treatment *X* time: *F*_(1,13)_ = 12.30, *p* = 0.004; effect of treatment: *F*_(1,13)_ = 0.04, *p* = 0.84; effect of time: *F*_(1,13)_ = 12.30, *p* = 0.004). We further found that STZ treatment showed a strong trend toward increased integrated density of BODIPY^493/503^ at the 12-week endpoint (*p* = 0.07), which was significantly different from the 6-week endpoint (*p* = 0.001) (Figure 10). In general, our findings demonstrated that lipid accumulation in VTA had a similar pattern with the IPN in the STZ group.

## Discussion

Diabetes has comorbidity with mood disorders, specifically anhedonia and depression (Sartorius, 2018; Wang et al., 2022), and is a risk factor for Parkinson’s and Alzheimer’s diseases (Stanciu et al., 2020; Wang et al., 2009; Labandeira et al., 2022), both of which are characterized by mood symptoms in early phases. The reward circuitry has a central role in the onset and progression of mood disorders in humans, which are recapitulated in animal models (Russo and Nestler, 2013). The MHb and IPN are two conserved regions of the reward circuitry that have been linked to both metabolism and mood, but little is known about their role in Alzheimer’s disease or their response to risk factors such as diabetes. Multiple studies have shown that the MHb-IPN participates in fear memory formation, nicotine addiction, and anxiety-like behavior (Zhang et al., 2016; Zhao-Shea et al., 2015; Cho et al., 2020). Understanding metabolic changes in the MHb and IPN during chronic hyperglycemia in an animal model of T1D could provide insight into the mechanisms underlying the correlation between diabetes and mood disorders. To examine the impact of diabetes on the MHb and IPN, we used an STZ model to induce a state of insulin depletion and chronic hyperglycemia, resulting in elevated blood glucose and elevated HbA1C at 6- and 12-week endpoints. Consistent with other reports, STZ-treated mice initially lost weight (−8.25 ± 5.19% baseline weight) at the 6-week endpoint, which recovered to near baseline by the 12-week endpoint (3.56 ± 3.30%) (Figure 1).

Chronic hyperglycemia in T1D is paralleled with an energy shortage in peripheral cells, as insulin deficiency leads to an inability to internalize glucose for use as an energy source (Matsuura et al., 2022; Hebert and Nair, 2010). Glucose is a major energy source for the brain; however, the brain does not generally require insulin for glucose uptake, despite the widespread expression of insulin receptors (Koepsell, 2020; Kleinridders et al., 2014). Therefore, it can be expected that the brain will be exposed to higher levels of glucose under states of hyperglycemia and that this change in glucose availability will modify the activity, structure, and function of the brain, particularly the mitochondria. In this study, we examined the mitochondrial response to hyperglycemia in the MHb and IPN in terms of distribution and morphology. We found that STZ-induced hyperglycemia increased mitochondrial numbers and occupied area in the vMHb specifically at the 6-week endpoint (Figure 2) and demonstrated coincident changes in complexity. Increasing mitochondrial number and elongation is a general response of the cell to energy status, typically associated with the lack of glucose uptake (Li et al., 2017; Ngo et al., 2023). In addition to the number and distribution, mitochondrial morphology is also affected by cellular energy levels. A higher AR in mitochondria represents an elongated structure, which leads to more ATP production and elevated oxidative phosphorylation in the cell (Yao et al., 2019). We found that mitochondrial AR and FF were increased in the MHb at the 6-week endpoint (Figure 4). Furthermore, we showed for the first time that glucose level and HbA1C positively correlate with mitochondrial numbers and morphology in the vMHb (Figure 5) at the 6-week endpoint. Together, these changes in mitochondrial number and morphology in the MHb and dIPN at the 6-week endpoint are largely consistent with observations *in vitro* when cells are exposed to increased glucose and confirm an increase in glucose available to the brain at subacute stages (6 weeks), but the increased FF suggest unique adaptations to hypoglycemia in the vMHb that are more typically associated with lower glucose levels. Interestingly, we found that by 12 weeks after STZ treatment, mitochondria in the vMHb returned to near baseline levels, suggesting compensatory adaptations. Indeed, it has been shown that chronic hyperglycemia results in the downregulation of glucose transport across the blood–brain barrier by limiting cerebral blood flow (Sanchez-Rangel et al., 2022; Hwang et al., 2019). Future studies should examine whether these compensatory adaptations occur at the level of the mitochondria or can be attributed to alterations in glucose delivery to the brain.

We next examined the IPN, which is the target of the MHb and contains extensive cholinergic axons from MHb neurons that contribute significantly to the volume of the IPN (Ables et al., 2023). We found that mitochondrial dynamics in the IPN mirrored those that we observed in the vMHb. Interestingly, we found that the dIPN was more prominently affected than the ventral IPN in terms of the number of mitochondria (Figure 3), with a significant increase in mitochondrial number at 6 weeks, while the vIPN did not demonstrate a significant change in the number but was more affected in terms of mitochondrial shape (Figure 6). In contrast to the vMHb, where mitochondria increased their AR and FF, in the vIPN, mitochondria were less complex. It is unclear from this study whether the mitochondrial changes in the IPN reflect the cholinergic axons from the vMHb or reflect the neurons and glia within the IPN itself. This is important to know to understand the potential implications of these findings. Should these findings largely reflect the effects of the cholinergic axons, this would further support our hypothesis that these neurons are vulnerable to hyperglycemic insult. The implications of this would support a scenario whereby diabetes-induced MHb dysfunction could contribute to early mood disorder symptoms in the course of Alzheimer’s disease.

One of the questions we sought to answer with this study was whether the cholinergic vMHb was more sensitive to hyperglycemia than other brain areas. To address this, we examined the adjacent LHb. Strikingly, the LHb did not show any significant changes in mitochondrial numbers (Figure 2). Together, our results showed that the MHb was more sensitive to hyperglycemia in terms of mitochondrial changes in distribution and morphology compared to the LHb and the IPN. One possible explanation could be that the MHb may be exposed to more glucose than the IPN, due to its direct communication with cerebrospinal fluid in the third ventricle, perhaps granting it the ability to sense metabolic challenges quickly. Our correlations also support this conclusion, as the IPN findings correlate only with chronic measures of glycemia, (HbA1C) but not blood glucose (Figure 7). In support of the ability to sense metabolic changes, the MHb is known to specifically express the orphan G-protein coupled receptor *Gpr151*, which functions to limit nicotine intake (Antolin-Fontes et al., 2020), and individuals from the UK Biobank cohort who are heterozygous for loss of function alleles of *Gpr151* have decreased risk of obesity and diabetes (Emdin et al., 2018). The MHb also expresses *Tcf7l2*, the most linked gene to diabetes risk, and plays a key role in conveying the risk of developing diabetes after heavy nicotine use (Duncan et al., 2019). Alternatively, the MHb is known to be tonically active (Gorlich et al., 2013), and action potentials require energy in the form of glucose, as does the synthesis of ACh. Finally, as we mentioned above, the changes in mitochondrial numbers and dynamics in the IPN may actually be reflective of the cholinergic axons from the vMHb and our ability to detect changes is limited with the approach that we utilized here. Future studies will be needed to understand how the MHb and IPN respond to chronic hyperglycemia in terms of neuronal firing and neurotransmission and the resultant behavioral consequences, as well as functional studies and higher resolution examination of the mitochondria in the IPN.

When glucose is in excess, it is stored as lipid droplets and when glucose is limited, mitochondria can produce energy through fatty acid *β*-oxidation. Therefore, given that we found altered mitochondrial numbers and morphology as a result of hyperglycemia, we expected to observe changes in LDs in the MHb and IPN. Surprisingly, we found that BODIPY^493/503^ integrated density, used as a proxy for LD number, was not different at the 6- or 12-week endpoints in the MHb (Figure 7), but was increased in the IPN at the 12-week endpoint (Figure 9). Mitochondria will associate with LDs when utilizing fatty acids as a fuel source (Benador et al., 2018), although we were not able to visualize this with our current approach. Despite this limitation, we were able to detect diabetes-induced changes in lipid distribution. To our surprise, these changes occurred in the LHb (Figure 7) and the VTA (Figure 10). Here, we found that lipid increased in the LHb at 6 weeks and decreased at 12 weeks, while the opposite pattern was observed in the VTA. Together, these results support the conclusion that each brain area responds to hyperglycemia with its own unique temporal and metabolic dynamics and that the brain is not uniformly affected.

Finally, our findings here demonstrate that both the brain and the body proceed through a series of stages in response to the onset and progression of diabetes. Like the time course of body weight (Figure 1), the mitochondria within the MHb and IPN mount a robust response to hyperglycemia at the 6-week endpoint that returns to near control levels by the 12-week endpoint. Our data support the idea that the response of the body to dysglycemia is not static, rather it seems to evolve over time, and the brain is no exception. While many studies have examined reward circuitry function in people with diabetes, very few studies have examined the longitudinal implications of diabetes on brain function. This is particularly surprising given that diabetes is an established risk factor for several neurodegenerative diseases. Future research should seek to understand what the long-term effects of diabetes on reward circuitry are, whether there are critical windows for intervention to reverse these changes, and to what extent these findings might be generalizable to the rest of the brain. Understanding the metabolic response of the MHb-IPN circuit to chronic hyperglycemia can open new therapeutic avenues for treating mood disorders in diabetic patients, or at the very least, provide more impetus for early screening and detection to prevent lasting damage to the brain.

### Limitations of the study

There are several notable limitations of this study. First, we did not examine female mice in this study. Female mice are resistant to STZ treatment and induction of diabetes in them is one of the challenges in metabolic research (Furman, 2021; Saadane et al., 2020). Second, the small sample size, (n = 4–5 per group) does not allow us to draw strong conclusions from the correlation analysis. Future studies with larger sample sizes and a wider distribution of blood glucose and HbA1c would strengthen these associations. Third, this study lacks markers of cellular specificity of the effects of STZ-induced diabetes on mitochondria and lipids, especially a cholinergic marker. Our approach using a single mitochondrial marker limited our ability to assign these effects to neurons or glial cells. Given that studies have demonstrated that mitochondria have different phenotypes in different cell types (Lopez-Fabuel et al., 2016), future studies should examine cell type-specific responses. Astrocytes are the main cells for glucose uptake in the CNS, but neurons express the insulin-dependent glucose transporter 3 (GLUT3) with high affinity to glucose uptake particularly at the synapse (Peng et al., 2021); therefore, analysis of astrocytic versus neuronal mitochondria under diabetic conditions would be of high interest. Finally, our confocal approach also did not allow us to examine the structure of individual mitochondria, including cristae which would be accessible with electron microscopy. It will be important to examine the cristae structure, which can reveal more about the functional state of mitochondria, in future studies.

## Data Availability

The raw data supporting the conclusions of this article will be made available by the authors, without undue reservation.

## Supplementary material

SUPPLEMENTARY FIGURE S1 Representative mitochondrial appearance at 2.5× zoom. Here we provide a closer view (zoomed in 2.5×) of representative processed mitochondrial images from each structure at each timepoint. VEH = vehicle; STZ = streptozocin; vMHb = ventral medial habenula; LHb = lateral habenula; dIPN = dorsal interpeduncular nucleus; vIPN = ventral interpeduncular nucleus.
