# Supplementary figures and images for: STZ-induced hyperglycemia differentially influences mitochondrial distribution and morphology in the habenulointerpeduncular circuit
Source: Front Cell Neurosci. 2024 Dec 23;18:1432887. doi: 10.3389/fncel.2024.1432887 (PMC11700986; doi:10.3389/fncel.2024.1432887)

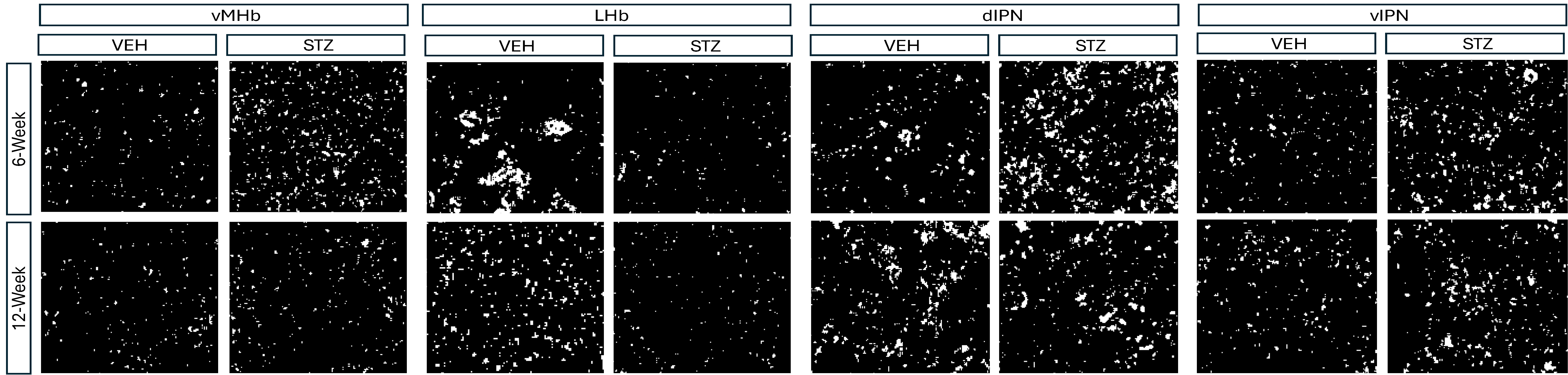

Supplement: SUPPLEMENTARY FIGURE S1 — Representative mitochondrial appearance at 2.5× zoom. Here we provide a closer view (zoomed in 2.5×) of representative processed mitochondrial images from each structure at each timepoint. VEH = vehicle; STZ = streptozocin; vMHb = ventral medial habenula; LHb = lateral habenula; dIPN = dorsal interpeduncular nucleus; vIPN = ventral interpeduncular nucleus. [file Image_1.tiff]
